# Adjusting *Aspergillus niger* pellet diameter, population heterogeneity, and core architecture during shake flask cultivation

**DOI:** 10.1186/s13068-025-02661-2

**Published:** 2025-06-12

**Authors:** K. Engelbert, C. Deffur, T. C. Cairns, F. Zhang, T. Kheirkhah, H. Winter, S. Junne, P. Neubauer, H. Briesen, V. Meyer

**Affiliations:** 1 Institute of Biotechnology, Chair of Applied and Molecular Microbiology, Technische Universität Berlin, Straße des 17. Juni 135, 10623 Berlin, Germany; 2 School of Life Sciences Weihenstephan, Chair of Process Systems Engineering, Technical University of Munich, Gregor-Mendel-Straße 4, 85354 Freising, Germany; 3 Institute of Biotechnology, Chair of Bioprocess Engineering, Technische Universität Berlin, Straße des 17. Juni 135, 10623 Berlin, Germany; 4 Department of Chemistry and Bioscience, Aalborg University, Niels Bohrs Vej 8, 6700 Esbjerg, Denmark

**Keywords:** *Aspergillus niger*, Macromorphology, Micromorphology, Pellet, Talc microparticles, Seed culture, Diameter distribution, Heterogeneity

## Abstract

**Background:**

Filamentous fungi form a range of macromorphologies during submerged cultivation including dispersed mycelia, loose clumps, and pellets. Macromorphological development is usually heterogenous, whereby mixtures form due to a complex interplay of growth, aggregation, and fragmentation. Submerged macromorphology strongly impacts product titres and rheological performance. Nevertheless, studies that systematically investigate the quantitative effect of cultivation parameters on macromorphology and heterogeneity are lacking.

**Results:**

In this study, we have developed shake flask cultivation conditions which enable reproducible macromorphological control of the multipurpose cell factory *Aspergillus niger*. Tested culture parameters included various spore titres, concentration of talc microparticles, shaking frequency, and presence/absence of baffles (*n* = 48 conditions). We quantified macromorphology (e.g., pellet diameter) using high-throughput two-dimensional image analysis and report intra-flask heterogeneity and flask-to-flask variation. These data identified optimal culture conditions which cause minimal macromorphological variation within individual flasks and between technical replicates. We demonstrate that pellet diameter can be reproducibly adjusted between experiments using simple cultivation conditions, and use these parameters to prove larger pellets secrete more protein while consuming less glucose. Linear regression models allowed us to identify spore concentration, shaking frequency, and talc concentration as crucial parameters impacting pellet diameter. Finally, we used a newly developed microtomography (µ-CT) approach to quantify the three-dimensional internal architecture for thousands of pellets at the cellular level. Cultivation conditions drastically impacted internal architecture. For the first time we report distinct types of pellets- those formed from a single (I) or multi-spore (II) core, and additionally pellets formed by agglomeration of mature pellets (III). Remarkably, these data show that a pellet of 2 mm consists of up to about 30 m of total hyphal length and contain approximately 200,000 tips.

**Conclusions:**

This study identifies simple methods for adjusting macromorphology and heterogeneity, which will enable facile testing of different macromorphologies for maximizing product titres. For the first time we have investigated how pellet internal architecture is impacted by numerous culture parameters. We propose a new pellet classification system based on internal spore core architecture, thus broadening our understanding of fungal macromorphological development and opening up new avenues for bioprocess or strain engineering.

**Supplementary Information:**

The online version contains supplementary material available at 10.1186/s13068-025-02661-2.

## Background

*Aspergillus niger* is a multipurpose cell factory used for the industrial production of biofuels, enzymes, organic acids, antibiotics, and secondary metabolites. In liquid cultivation, *A. niger* can have a dispersed, pelleted, or heterogenous mixtures of both morphologies [1, 2]. This significantly influences product formation capacity for a range of desired molecules [3]. Pelleted morphologies seem to be beneficial for production of secondary metabolites and organic acids [4, 5]. For protein production, several studies point out that the pellet diameter plays a critical role [6, 7]. Consequently, pellet populations are primarily described by their diameter [8–10]. However, the formation of pellet populations is often accompanied by heterogeneity in pellet shape (e.g., aspect ratio, surface structure) [11]. Additionally, recent microtomography (µ-CT) imaging of the pellet core [23] has revealed heterogeneity also occurs within individual pellets e.g. in distinct internal regions with varied hyphal density/branch rates. This causes changes in diffusion of nutrients and oxygen to the central part [12–14]. The situation is further complicated by extensive variation between technical replicates in shake flask cultivations. Indeed, where researchers require highly reproducible macromorphologies between technically replicated cultivation, it is normally necessary to scale up to fully controlled bioreactor fermentation, which greatly limits throughput [15, 16].

Mechanistic explanations for individual pellet, intra-flask, or flask-to-flask heterogeneity are severely lacking for *A. niger* and other filamentous fungi. In submerged cultivation, *A. niger* forms pellets through coagulative spore agglomeration whereby spores initially cluster together, then germinate and develop hyphae. As growth continues, further agglomeration occurs, ultimately developing a mature pellet [1]. The first aggregation step is mainly driven by electrostatic and hydrophobic interactions between the spores, while in the second step spore wall components interact more specifically [17]. It has been confirmed that the basic hyphal architecture of *A. niger* pellets develops during the first and second agglomeration steps and is maintained throughout the rest of the growth period [18]. The first aggregation step normally occurs within 6 h after inoculation, while the second agglomeration step is completed after approximately 14 h [19].

It is generally accepted that variations in agglomeration, hyphal growth, and pellet fragmentation between different strains or cultivation conditions impact macromorphology and heterogeneity at the individual pellet, population or replicate level [3]. Morphological engineering (e.g., changes in pellet diameter or shape) can be achieved through genetic modification of strains [2, 9, 20], for example by conditional expression or deletion of genes required for normal hyphal branching [21], vesicle trafficking [22], signalling cascades [4], or cell wall composition [23]. Additionally, adjustments in media composition (e.g., carbon source, ion availability, pH [13, 16, 24]) or process parameters (agitation speed, spore concentration, microparticle addition [24]) have extensive impacts on submerged macromorphology and heterogeneity [1, 25–28].

Taken together, it is clear that reproducible control of macromorphology and heterogeneity in submerged culture is an important objective for industrial applications of filamentous fungal cell factories. However, studies linking fungal macromorphology, heterogeneity, and productivity often only study the impact of a single gene, media component, or process parameter [11, 27, 29–31]. We hypothesise that this significantly limits our ability to understand and control submerged fermentation.

This work aims to bridge the gap by investigating the relationship between multiple cultivation parameters with pellet diameter, population heterogeneity, and flask-to-flask variation at both the macroscopic and micromorphological levels. Given that productivity is likely dependent on an integrated system of process conditions, macromorphology, and heterogeneity, understanding these dynamics is crucial to maximally harness fungal productivity. This study thus provides a foundation for obtaining a targeted morphology in shake flasks, serving as a starting point for strain engineering, process development, and scale-up.

## Materials and methods

### Strain and spore solution preparation

The recombinant strain ÖV4.10 [32] was used for this study. Spores were inoculated on complete medium (CM) [33] in Petri dishes, grown at 30 °C for 5 days and harvested and prepared on the day of use as previously described [34].

### Shake flasks cultivations

Shake flask cultures were grown for 24 h to study pellet populations or 16/20 h for studying growth behaviour (cell dry weight (CDW)/glucose consumption). 50 mL CM cultures were inoculated to a final concentration of 5 × 10^6^, 5 × 10^5^, and 5 × 10^4^ spores mL^−1^ and experiments were conducted in 250 mL flasks which were either equipped with 2 baffles or were unbaffled. Cultures were incubated at 30 °C. Where indicated, CM media was supplemented with talc microparticles (Sigma-Aldrich, 350 mesh, *x*_10,3_ = 1.93 µm, *x*_50,3_ = 5.12 µm, *x*_90,3_ = 10.08 µm) in concentrations of 0, 1, 5, 10 g L^−1^. Talc particle size was analysed using a HELOS/KR equipped with a R1 lens (Sympatec GmbH, Germany). Cultures were grown in Infors HT Multitron Standard shaker (Infors AG, Switzerland) with an amplitude of 25 mm at constant shaking frequency of 150 rpm or 250 rpm. CDW was determined by filtering 10 mL culture broth as described by [34]. The amount of talc in 10 mL medium (1, 5, 10 g L^−1^) was similarly determined and subtracted from the CDW. Glucose consumption and total protein was analysed as described previously [23].

### 2D image analysis for macromorphological analysis

Macromorphology was analysed by using images obtained with a Leica S8APO stereomicroscope (Leica Microsystems GmbH, Germany) connected to a Leica MC120 HD camera (Leica Microsystems GmbH, Germany). For this purpose, defined amounts of culture broth and physiological saline solution (0.89% (wv^−1^) NaCl) were pipetted into an empty Petri dish as well as 2 µL of 50% (v/v) Tween 20 to reduce surface tension. The solution was swirled gently and images were taken as described before [35]. Since the pellets from most cultivation conditions did not exhibit clear spore agglomerates, a new method was derived from the image analysis pipeline of [35] to detect the pellets in shake flasks. The pellets were segmented based on the red channel of the images (Figure S1b), as hyphae in this channel show a good contrast to the background [35] and most impurities show a weak signal. The first step applied an opening by reconstruction (MATLAB function “imerode” and “imreconstruct”) to the red channel image (Figure S1c) with a structured element of 10 pixel to remove the noise object without damaging the pellet structure [36]. Then similar operations for closing by reconstruction (MATLAB function “imdilate”, and “imreconstruct”) with a structured element of 5 pixel were performed (Figure S1d). From the resulting filtered and smoothed grey-level image of the red channel, an image with the location of the regional grey-level maxima representing the central pellet regions (Figure S1e) was extracted (MATLAB function “imregionalmax”), and the mask of the pellets was obtained by binarization (Figure S1f). This pellet mask reflects the contour of the pellets accurately. Next, to determine the boundary between closely spaced pellets, marker-controlled watershed segmentation [37] (Figure S1i-j) was applied to the Euclidean-distance-transformed pellet mask (Figure S1g), with the grey values of the central pellet regions set as minima (MATLAB function “imimposemin”) (Figure S1h). During post-processing, the pellets intersecting the image border could introduce distortions into the statistical results and were consequently excluded (Figure S1k). Furthermore, pellets falling outside the predefined diameter range of 100–3000 µm were considered less likely to represent single fungal pellets and were removed (Figure S1k). The entire process as illustrated in Figure S1a-l was performed twice for each sample.

Pellets that could not be analysed with the adjusted 2D image analysis pipeline (15%), due to either too large diameter or image quality, were analysed manually by using Fiji (ImageJ) version 1.54 [38]. For this, the pixel to µm ratio was determined for each condition and the scale set globally. The pellets’ area was determined by circling each individual pellet and used to calculate the area equivalent diameter (Figure S2).

### Characterisation of pellet populations

For the characterisation of pellet populations, data derived from 2D image analysis were used. Populations with less than 30 analysed pellets were considered as poorly grown and therefore excluded from further calculations of population characteristics.

To ensure robustness against extreme values and suitability for differently shaped unimodal distributions, the pellet diameter of a population is represented by the median.

In this study, population heterogeneity is defined as the range of pellet diameters within a population represented by the interquartile range (IQR) between the 25th and 75th percentile. Thus, it describes the spread of the middle 50% of the population.

The flask-to-flask-replicate variance was determined by calculating a distribution-free overlap coefficient (OVL) [39] of the number-density distributions of pellet diameter populations (q_0_), describing the number of pellets per diameter class (= histogram bin), normalised to the total pellet number. The OVL measures the overlap of two defined sets (A, B) by how much of the smaller set is a subset of the bigger one, and ranges from zero (no overlap) to 1 (smaller set is found entirely in the larger set). Note that due to normalisation, both distributions integrate to 1 and the distinction between populations with high and low pellet numbers is irrelevant in practice. The OVL is defined as followes:

$$\text{OVL} \left(A,B\right)= \sum_{i=1}^{80}\text{min}({f}_{A}\left({d}_{i}\right), {f}_{B}\left({d}_{i}\right))$$

where ${f}_{A}(d)$ and ${f}_{B}(d)$ are the q_0_ distributions for two defined sets A and B, respectively. The overlap between the two distributions is quantified by the intersection size, which represents the summed overlap across all diameter classes ${d}_{i}$. For each replicate, the q_0_ distribution of particle diameters was computed for the range 0 to 4000 µm, using 50 µm bin size (= 80 diameter classes). Since the overlap coefficient is defined for pairwise comparison, it was calculated for all replicate pairs. It was set to zero for populations with less than 30 data points overlapping between the respective replicate combinations . The final similarity measure was obtained by averaging the pairwise OVL values across all replicates.

### Multiple linear regression analysis

A multiple linear regression analysis was performed using MATLAB (Version R2023a) with the function ‘fitlm’. The independent variables were spore concentration, talc concentration, shaking frequency, and the presence or absence of baffles. Their influence on the dependent variable, the median pellet diameter, derived from 2D image analysis, was investigated. For multiple regression analysis to yield meaningful results, a few basic assumptions should be met: relationships are linear; residuals (prediction errors) are independent; residuals are evenly spread—a property called homoscedasticity—and residuals are roughly normally distributed. For more details, see [40]. Assumptions were assessed using simple graphs[41]: a residual plot shows whether the residuals form a random cloud around zero – if they do, linearity, independence, and homoscedasticity are likely satisfied. Normality is assumed when the histogram is bell-shaped and the QQ-plots fall roughly on the diagonal line. If these criteria are met, adjusted R^2^ can be a further characteristic of the quality of the model.

To ensure that the significant independent variables are not correlated with each other (multicollinearity) and contribute unique information to the model, two tests were run. Firstly, a correlation matrix [42] was calculated using the MATLAB function “corr”. While 1/-1 represent a strong positive/ negative pairwise linear correlation, a value of zero indicates no correlation. Secondly, the Variance Inflation Factor (VIF) was calculated as described in [42] to confirm that no single predictor showed excessive overlap with the others.

### 3D image analysis for micromorphological analysis

For micromorphology analysis, pellets from shake flask cultivations were freeze-dried following the procedure described previously [23]. Imaging was conducted by synchrotron microcomputed tomography at the Deutsches Elektronen Synchrotron (DESY) using the beamline P05 of PETRA III in Hamburg, Germany as previously described [18]. As multiple pellets were present within the pellet holder during the 3D measurement to maintain high throughput, an automatic segmentation of individual pellets from the 3D images was performed using a watershed transformation. However, due to the diverse morphology and diameter of the pellets, the separation was not always accurate, resulting in cropped images containing multiple pellets instead of one. For these, further morphological analyses could not be accurately conducted. Consequently, based on manual examination of the mean intensity projection (meanIP) images, decisions were made regarding whether to proceed with an analysis or to exclude those incorrectly segmented pellets from further analysis.

## Results

### Adjusting *A. niger* pellet populations in shake flask cultivation is possible by altering simple process parameters

Shake flask cultivation is by far the most common approach for early process development and preliminary assessments of filamentous fungal productivity [43, 44]. We therefore modified simple and cost-effective cultivation parameters known to impact macromorphology/heterogeneity in shake flasks [27, 45–47]. This included final spore concentrations of 5 × 10^4^, 5 × 10^5^, or 5 × 10^6^ mL^−1^, low (150 rpm) or high (250 rpm) shaking frequency, addition of 0, 1, 5, or 10 g L^−1^ talc, and flask design (with or without baffles). This led to 48 cultivation conditions from which macromorphology was quantified in technical triplicates after 24 h of cultivation. For simplicity, each cultivation condition was denoted using an index number (Table 1).

**Table 1:** Simple index system used to denote cultivation parameters in this study. An example index is: S6-AH-T0-B1, i.e., cultivation was carried out with 5 × 10^6^ spores mL^−1^, 250 rpm (= high), 0 g L^−1^ talc, and a baffled flask

Parameter	Index denotation	Qualifier	Note
Spore concentration	S	4	5 × 10^4^ spores mL^−1^
		5	5 × 10^5^ spores mL^−1^
		6	5 × 10^6^ spores mL^−1^
Agitation	A	L	150 rpm
		H	250 rpm
Talc microparticle concentration	T	0	0 g L^−1^
		1	1 g L^−1^
		5	5 g L^−1^
		10	10 g L^−1^
Flask baffles	B	0	Non-baffled flask
		1	Baffled flask

**Fig. 1:**
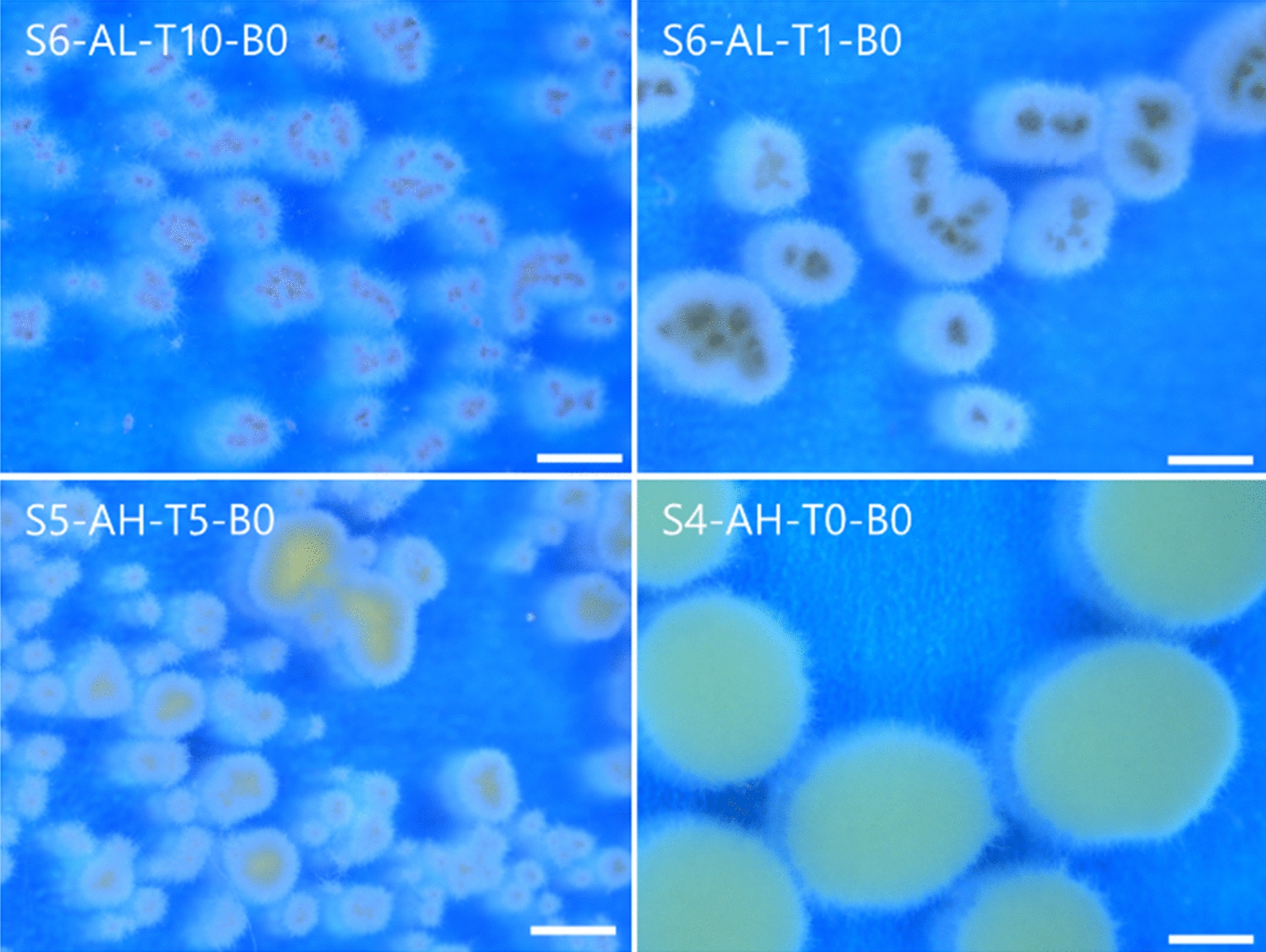
Pellets from selected cultivation conditions with varying appearance and diameter. *A. niger* was cultivated in 48 shake flask conditions and pellet populations were quantified. The cultivation parameters inoculation spore concentration (S), shaking frequency (A), different talc concentrations (T), and flask form (B) were varied (see Table 1). Images were taken after 24 h. The scale bar represents 250 µm

**Fig. 2:**
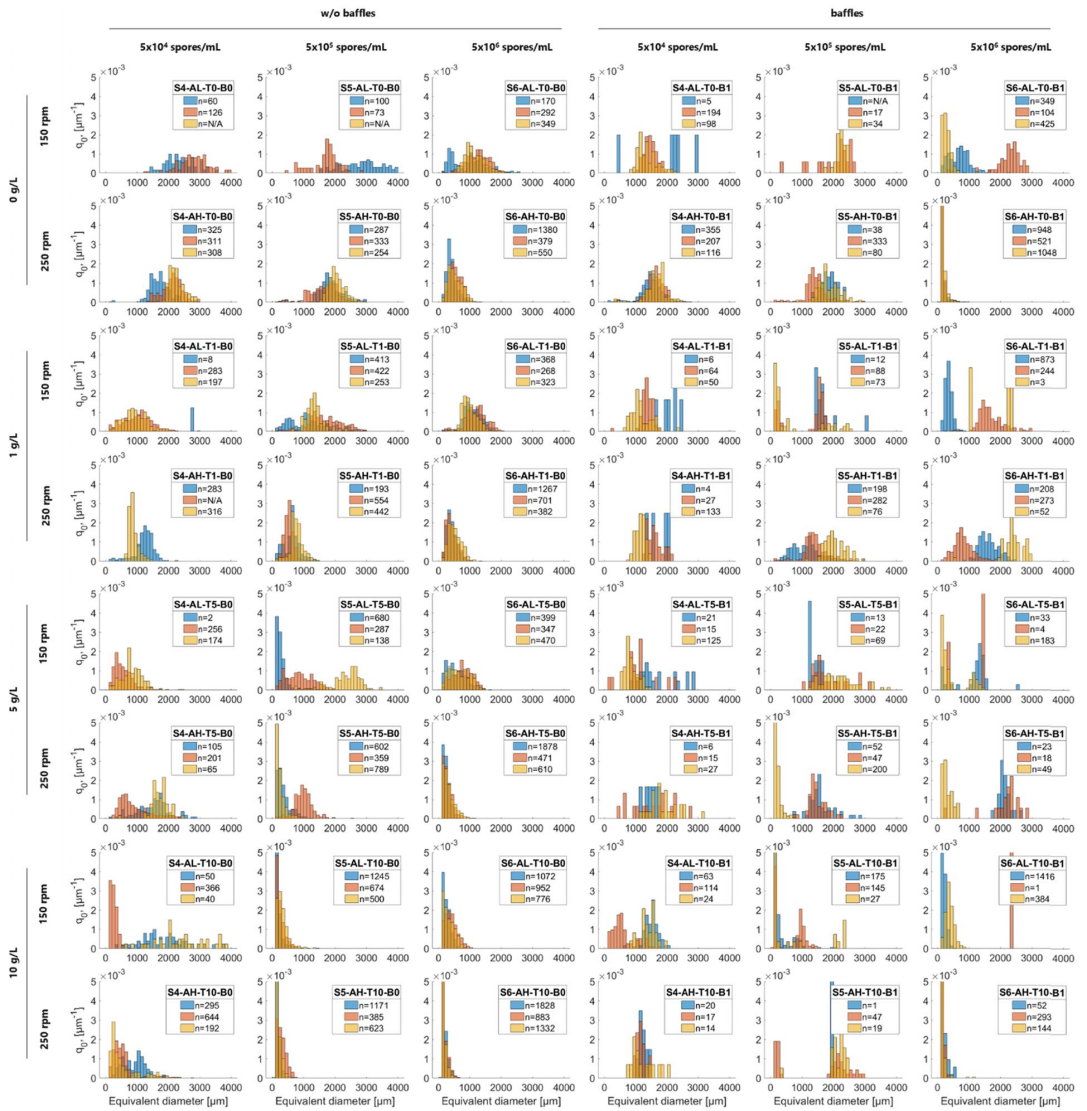
Pellet diameter distributions of 48 cultivation conditions of *A. niger*. The x-axis shows the measured area equivalent pellet diameter (ED) and the y-axis the number-density distribution of the pellet populations (q_0_), describing the number of pellets per bin, normalised to the total pellet number. The colours blue, red and yellow represent biological replicates grown from independent spore solutions with “n” being the number of analysed pellets for each condition and “N/A” when analysis was not applicable. To improve readability, the highest values have been partially cropped (x- and y-axis), the bin size is 100 µm. Populations can exhibit a unimodal number-density distribution (e.g. S5-AH-T1-B0, S6-AH-T0-B1) having one peak or a multimodal number-density distribution (e.g. S5-AL-T1-B1, S5-AL-T1-B1) characterised by multiple distinct peaks

A total of 44,000 pellets were analysed using a simple, high-throughput image analysis approach (Fig. 1 and supplemental Figure S3a-c). Since the pellet diameter plays a crucial role in productivity, we used the area equivalent pellet diameter (equivalent diameter, µm) from the 2D images to quantify macromorphology. Pellet homogeneity within individual flasks was quantified by calculating the interquartile range (IQR) of the pellet diameter distribution (Fig. 2 and Figure S5). Finally, flask-to-flask variation was determined by plotting pellet diameter histograms for technical replicates (*n* = 3), and subsequently calculating the distribution-free overlap coefficient (OVL) of the histograms (Fig. 2 and Figure S6).

In these experiments, pellets of *A. niger* were almost always spherical. In four conditions with high agitation (S6-AH-T10-B0, S6-AH-T5-B0, S6-AH-T0-B1, S5-AH-T10-B0), a yellow pigmentation was observed (Figure S3a-c). It should be noted that initially turbid media became clear in the majority of conditions with talc microparticles after 24 h, suggesting that nearly all the talc particles were integrated into the pellets (Figure S4). Median pellet diameters varied drastically, with diameters ranging from 134 ± 3 µm (S6-AH-T10-B1) to 2616 ± 326 µm (S4-AL-T0-B0, Fig. 2). In addition, differences in flow behaviour were observed when the cultures were swirled, whereby smaller pellets generally resulted in a more viscous liquid compared to larger ones (data not shown). Overall, these data demonstrate that the simple parameters tested can significantly alter the macromorphological development of *A. niger*.

Larger pellets with a higher median diameter, generally displayed greater heterogeneity, also represented by a higher IQR (Fig. 2 and Figure S5). Small pellets were mostly produced with high spore concentrations and with addition of 5–10 g L^−1^ talc (S6-AH-T10-B0, S6-AL-T10-B0, S5-AH-T10-B0, S5-AL-T10-B0, S6-AH-T5-B0, Fig. 2) suggesting that these conditions are a straightforward way to minimize pellet diameter and thus variation.

Regarding the flask-to-flask variation, non-baffled flasks with high spore concentrations produced the most uniform macromorphologies as determined by OVL (Fig. 2 and Figure S6). Conversely, low spore concentrations in baffled flasks tended to result in greater variability in cultivation outcomes among the replicates (Fig. 2 and Figure S6). Indeed, baffles appeared particularly critical, as cultivation conditions resulting in high technical reproducibility in standard flasks were poorly reproducible when baffled flasks were used (e.g., S6-AH-T5-B0/1, S6-AH-T1-B0/1, S5-AL-T10-B0/1, S5-AH-T1-B0/1, Fig. 2, Figure S6). Additionally, replicates with smaller pellet diameter generally resulted in lower flask-to-flask variance compared to cultures with larger pellets (Figure S6).

We thus suggest that cultivation conditions which result in high flask-to-flask variance (e.g., S6-AH-T1-B1, S6-AL-T0-B1, S5-AL-T5-B0, Fig. 2 and Figure S6) are broadly unsuitable for assessing fungal productivity due to low technical reproducibility of experiments. Additionally, for many of the 48 conditions (e.g., S6-AH-T5-B1, S6-AH-T1-B1, S6-AL-T1-B1, S6-AL-T0-B1, S5-AH-T5-B1, S5-AH-T5-B0, S5-AL-T5-B0, S5-AL-T0-B0, S4-AL-T10-B1, S4-AL-T10-B0, S4-AH-T5-B0), at least one replicate is shifted in pellet diameter distribution, indicating that these conditions may also be unsuitable for productivity assays (or at the very least require more than three replicates for robust and accurate analysis).

In total, 15 cultivation conditions were visually observed to have low flask-to-flask variation, which was substantiated by subsequent quantitative analyses (i.e., OVL ≥ 0.60, Figure S6). These conditions spanned a diverse range of pellet sizes, from very small (S6-AH-T0-B1, 134 ± 3 µm) to large pellets (S5-AH-T0-B0, 1841 ± 165 µm). We thus hypothesized that these conditions can be used to reproducibly adjust pellet diameter between shake flask experimental cohorts during productivity assays.

### Adjusting pellet diameter using simple cultivation parameters impacts glucose consumption and protein secretion

**Fig. 3:**
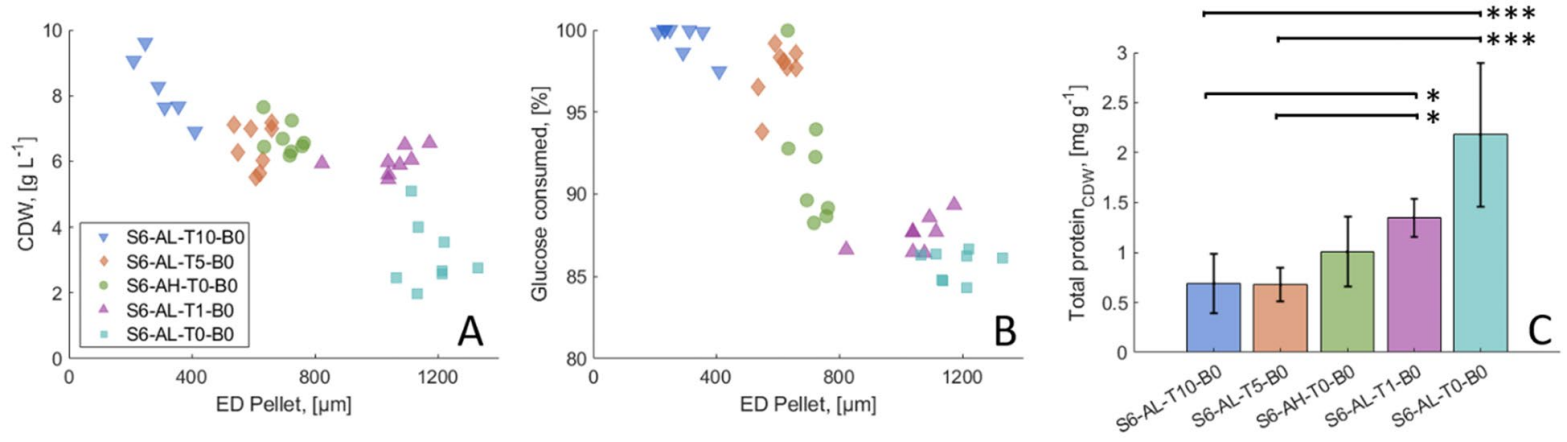
Growth behaviour is pellet diameter dependent. Cell dry weight (CDW) (**A**) and glucose consumption (**B**) were analysed after 16 h for five pellet populations of different diameters. Each population was cultivated in replicates (*n* = 8). The populations’ pellet diameter is represented by the median of the area equivalent diameter (ED) of each pellet population. **C** The total protein in the supernatant was measured for each replicate and normalised to the CDW. The post-hoc test with Bonferroni correction was used to perform pairwise comparisons between normalised total protein, with *p* < 0.05 (*) and *p* < 0.0005 (***)

Testing strain productivity from different macromorphologies with high reproducibility is an important goal of fungal biotechnology. We therefore selected five cultivation conditions which resulted in unimodal pellet populations and low flask-to-flask-variance (S6-AL-T10-B0, S6-AL-T5-B0, S6-AH-T0-B0, S6-AL-T1-B0, S6-AL-T0-B0, Fig. 2) and analysed these in a productivity screening. Each experimental cohort (i.e., cultivation condition) was tested using eight technical replicates. Cell dry weight (CDW) and glucose consumption were quantified across a 20 h time series (Figure S7) and total protein production was determined after 16 h of growth (Fig. 3).

Pellet diameter, CDW, and glucose consumption were reproducible in each experimental cohort, indicating the respective cultivation condition is indeed appropriate for productivity screening (Fig. 3, Figure S8). When the normalised glucose consumption (relative to initial concentration) was plotted against the pellet diameter, it became evident that the pellet diameter influences the rate of glucose consumption (Fig. 3 and Figure S7). Specifically, smaller pellets consumed glucose faster than larger ones, likely due to differences in their volume-to-surface ratio. In larger pellets, nutrient uptake could additionally be limited by the diffusion rate within the pellet structure. As a result, within the same time frame, smaller pellets achieve a higher maximum biomass than larger ones (Fig. 3 and Figure S7).

As different populations are at varying metabolic stages at 16 h, total protein production titres in the supernatant were normalized to the CDW. We were thus able to show that the population with the smallest pellet diameter also produced the lowest amount of total protein with 0.69 ± 0.30 mg g^−1^, while the largest pellets reached titres of 2.18 ± 0.72 mg g^−1^. The total protein analysis thus supports the hypothesis that different populations consistently produce a reproducible amount of total protein. While larger pellets secrete significantly more protein than smaller ones based on the CDW, controlling pellet diameter can be a key objective for process optimisation.

In summary these data confirm that future *A. niger* productivity screenings can be tested in the context of several user desired pellet diameters without compromising technical reproducibility by following the simple culture parameters detailed in Fig. 3.

### Linear regression approaches can be used to predict pellet diameter from cultivation conditions, but flask-to-flask variance is a major limitation

Multiple linear regression is a widely used tool for exploring linear relationships and understanding the influence of multiple variables on a target outcome. In this study, we used linear regression to assess the relationships between spore concentration, talc concentration, shaking frequency, and the presence or absence of baffles (independent variables) and median pellet diameter (dependent variable). Additionally, we aimed to develop a model as a tool for estimating the pellet diameter under a given set of cultivation parameters.

The model was based on the median pellet diameter from 2D image analysis, with each shake flask replicate (Fig. 2) included as a separate observation to capture variance. Multimodal populations (characterised by several distinct peaks) and those with less than 30 analysed pellets were excluded, resulting to a total of 107 replicates from 43 different cultivation conditions with diameters between 131 and 2996 µm (supplementary material 2).

An initial model approach ran into two problems: a high error variance (heteroscedasticity), evident from the funnel shape in the residual plot (Figure S9) and the deviation from normality of the residuals (Figure S9). Both issues are commonly encountered when the distribution of the dependent variable (pellet diameter) is far from beeing normal[48]. In our 107 observations, most conditions had small pellets, so the distribution was highly skewed (Figure S10). A common way to overcome this is to transform this variable before modelling [41]. Using natural logarithm (ln)-transformed pellet diameter values resolved both problems (Figure S11), indicating a better model fit. Additionally, the adjusted *R*^2^ value increased from 0.41 to 0.51. In a next  step, the independent variables spore concentration (50,000–5,000,000 spores mL^−1^) and shaking frequency (150–250 rpm) were ln-transformed, resulting in ranges of 10.82–15.42 and 5.01–5.52, respectively. This can make the relationship between the dependent and independent variables more linear and put variables on a similar scale [49]. As a result, the normal distribution of the residuals was further optimised (Figure S12). Having the assumptions for the multiple linear regression largely met, ensuring a reasonable balance between simplicity and accuracy, it was demonstrated that the independent variable ‘baffle’ had no significant effect (*p* > 0.05) and could be excluded from the model (Table S1). The other independent variables (spore concentration, shaking frequency, talc concentration) were identified as having a significant reducing influence (*p* < 0.01) on the pellet diameter (Table S1). This is consistent with findings in literature and also observed in this study (Fig. 2). To confirm that these variables acted independently, multicollinearity was investigated as described before. The correlation matrix (Table S2), showed no strong linear relationships between the independent variables. Consistently, the variance inflation factor (VIF) values were below the critical threshold of 10: 1.0000 for spore concentration, 1.0001 for shaking frequency, and 1.0001 for talc concentration. This indicates that multicollinearity is not an issue and that each variable contributes independent information to the model.

**Fig. 4:**
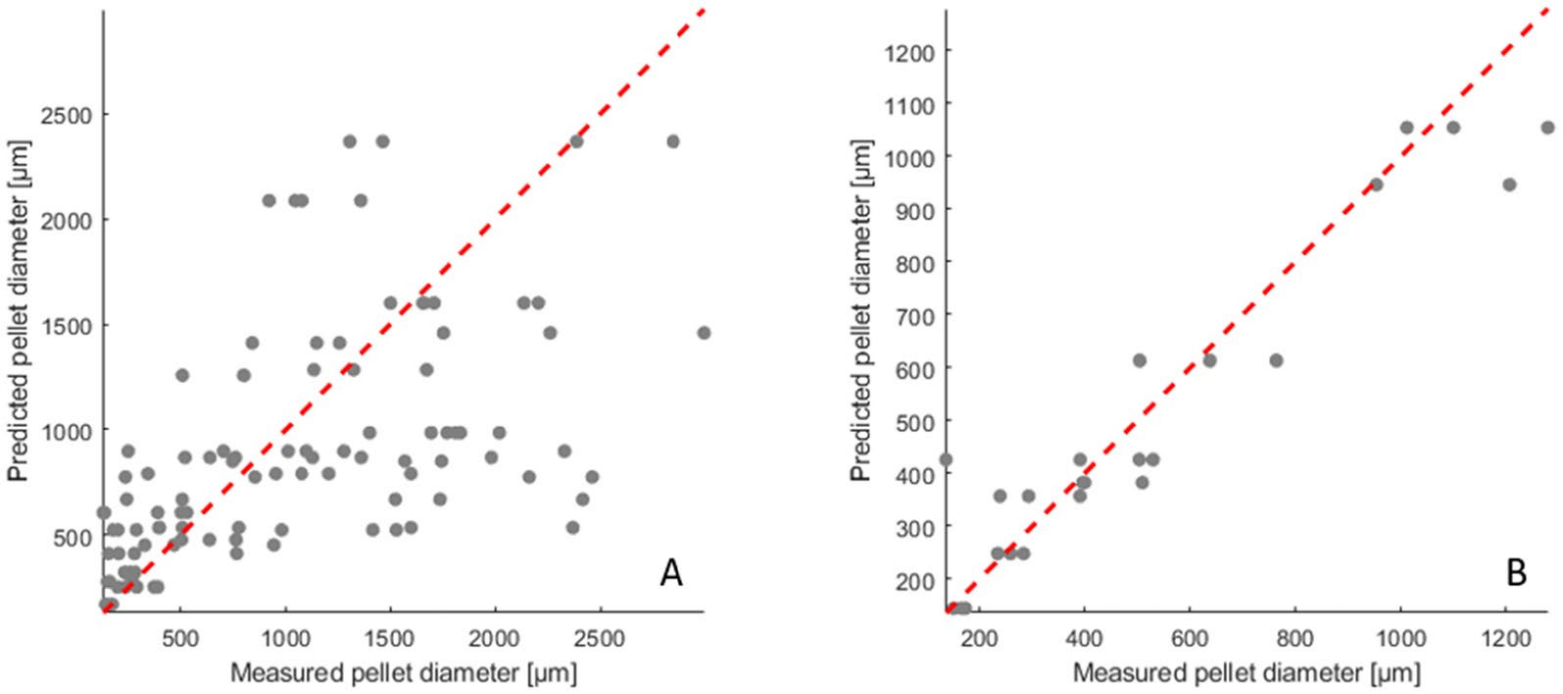
Parity plots comparing the calculated and experimental pellet diameters. The dashed red line represents perfect correlation (*y* = *x*). **A** Displays results for the comprehensive data set, consisting of 107 data points that also include data with a high variance between replicates. **B** Presents a subset of 24 data points, focusing on the most reproducible conditions, specifically those with the highest spore concentration and cultivation in non-baffled flasks. During model development, pellet diameters were ln-transformed and subsequently back-transformed for visualization

To assess the model, the calculated and experimental pellet diameters were compared in a parity plot (Fig. 4A). As reference, the perfect correlation (y=x) of a model with 100 % prediction accuracy is shown as a red line [48]. The noticeable scattering of the 107 data points, particularly for pellet diameters greater than 1200 µm, limits the achievable precision of the model and presumably contributes to the 49% of the variability in pellet diameter remaining unexplained.

However, for a useful tool, a data set with low variance between replicates is needed. Thus, a multilinear regression was performed using a data set of 8 specific conditions (S6-AL/H-T0/1/5/10-B0), focusing on the highest spore concentration (5 × 10^6^ spores mL^−1^) and non-baffled flasks, resulting in a total of 24 data points (supplementary material 2). By reducing the dataset to a smaller but higher quality subset with fewer input parameters, the linear regression model achieved an adjusted R^2^ value of 0.80 (Figure S13 and TableS3). This indicates that, for non-baffled flasks with the highest spore concentration, most of the variance in pellet diameter can be explained by only two process parameters. The parity plot of this reduced data set (Fig. 4B) shows good agreement between predicted and observed values from 150 to 1200 µm. This suggests that the reduced model is suitable for the prediction of the pellet diameter in that range using the independent variables agitation frequency (*A* in rpm) and talc concentration (*T* in g L^−1^). Since both, pellet diameter (*PD)* and talc concentration (*T)* were ln-transformed, the predicted pellet diameter can be obtained by back-transforming *PD*:

$$\text{PD} (\mu m)= {e}^{\left(15.863-1.7769 \cdot \text{ln}\left(A\right)-0.10856 \cdot T\right)}$$

During the evaluation of the linear regression model, it became evident that the variance between the replicates hinders the development of accurate mathematical models for predictive optimisation. While certain combinations of process parameters appeared more stable than others, the underlying causes of instability in other conditions could not be determined within the scope of this study. Further investigation, e.g. through more advanced modelling approaches, may provide new insights into process optimisation.

### *A. niger* pellets can be classified into three distinct core types

Recent developments enable the analysis of pellets at a micromorphological, three-dimensional level by using synchrotron radiation-based µ-computed tomography (SR-µCT) [18]. This technology has already been successfully applied to analyse the microparticle-enhanced cultivation (MPEC) of *A. niger* pellets [50]. Therefore, we hypothesized that employing this non-invasive method would provide valuable insights into the pellet formation process under different cultivation conditions.

For the micromorphological 3D-analysis pellets from non-baffled flasks were analysed originating from an additional fourth replicate specifically cultivated for the examination of micromorphological structures. The volume equivalent pellet diameter distribution obtained from 3D data was compared to the area equivalent diameter derived from the 2D-data. This comparison is shown in Figure S14. The results indicate that the fourth replicate is comparable to those used in the 2D analysis, suggesting that the population analysed with SR-µ-CT effectively represents the overall population, even with a lower number of pellets. This supports the validity of using the 3D data for further conclusions regarding pellet populations across different conditions.

**Fig. 5:**
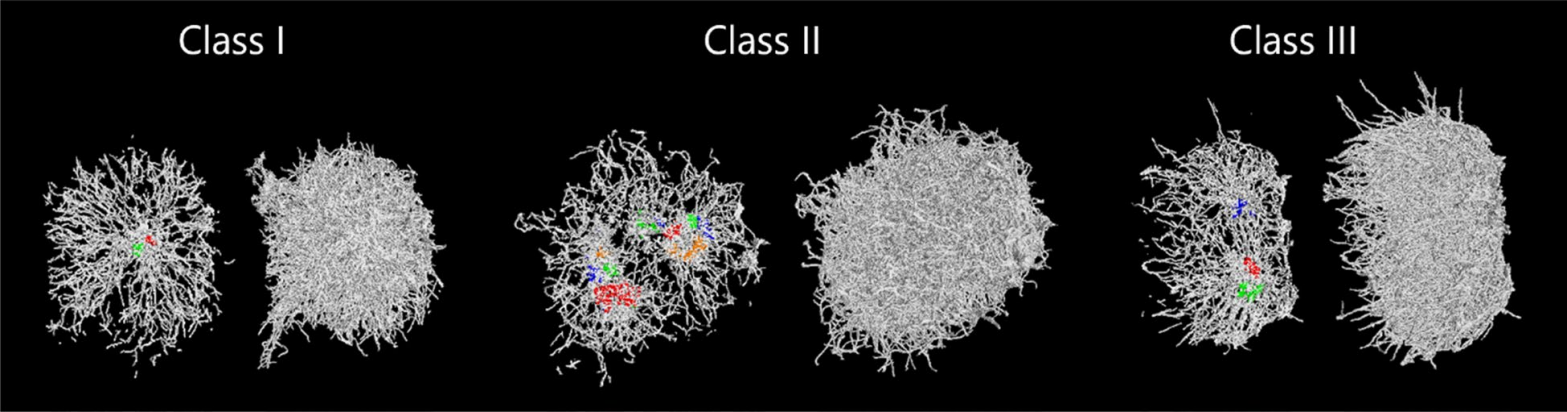
Pellet classification based on inner structure. The images obtained from SR-µ-CT measurements depict a 25 µm-thick cross-section through the centre of mass of the pellet (left) created by 3D rendering of the hyphae and talc (if applied). Spore clusters were calculated by a density-based clustering algorithm ("dbscan", MATLAB) with a minimum of 100 spores. For enhanced visibility, the calculated spore clusters of the presented cross-section are shown in different colours and the spores are enlarged by a factor of 9. Additionally, the outer morphology of each pellet type is presented for comparison (right). The images were created using VGSTUDIO MAX (version 3.2; Volume Graphics GmbH)

The analysis of over 2500 pellet structures revealed distinct differences, allowing classification into three distinct classes (Fig. 5). Class I pellets have a single, central spore core from which hyphae grow outwards. Such outward growth is generally radiant, although some sectoring may occur where hyphae are absent. Class II pellets display multiple spore cores which are not necessarily located at the centre of the structure. Hyphal growth from each core occurs towards the pellet periphery in a radiant way, although growth towards other spore core(s) is clearly reduced or absent. With regards to diameter, class II pellets are larger than class I. Finally, class III pellets appear to have initially existed as separate entities that fused during a later growth phase through the entanglement of their outer hyphae. It is important to note that agglomeration was not limited to “whole” pellets, but also the fusion of apparently separated mycelial fragments or “torn pellets”, resulting in pellets with elongated or asymmetrical shapes (Figure S15).

## Culture parameters impact pellet cores

**Fig. 6:**
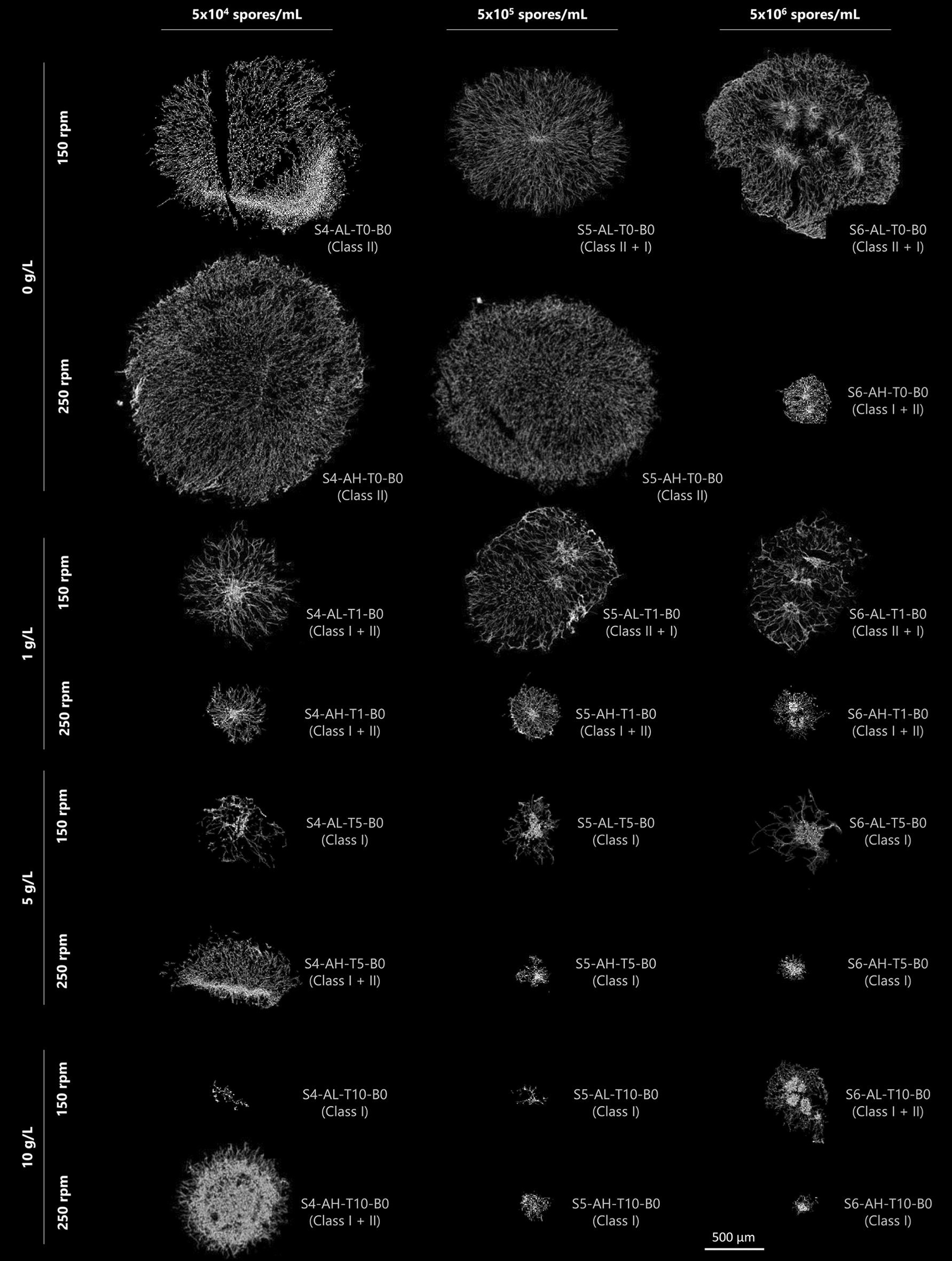
Grey level images of representative pellets from non-baffled shake flask conditions derived from synchrotron radiation-based µ-CT measurements. The images show mean intensity projections of a 25 µm-thick cross-section through the pellet’s mass centre. For each condition, the assignment of Class I, Class II and mixtures is indicated for orientation purposes, it does not represent a strict categorisation.  The g L^-1^ values indicate the talc concentration used in each cultivation condition

We reasoned that certain culture parameters tested in this study (Fig. 2) could impact internal core architecture. Over 2500 grey-scale images (exemplar given in Fig. 6) were studied and the associated SR-µ-CT data analysed. This analysis enabled the comparison of key structural metrics related to the internal architecture, such as the solid fraction, describing the percentage of hyphae and talc—if embedded—in the pellet volume, the number of spore cluster per pellet, and number of branches, providing deeper insights into the internal architecture of pellets in liquid cultures.

Interestingly, the addition of talc microparticles improved the visibility of spore clusters in the grey scale images of the mass centre, as they were mostly embedded during the spore agglomeration process (see Figure S4). The majority of the 2500 analysed pellets consisted of either Class I or Class II, while Class III pellets were observed less frequently.

**Fig. 7:**
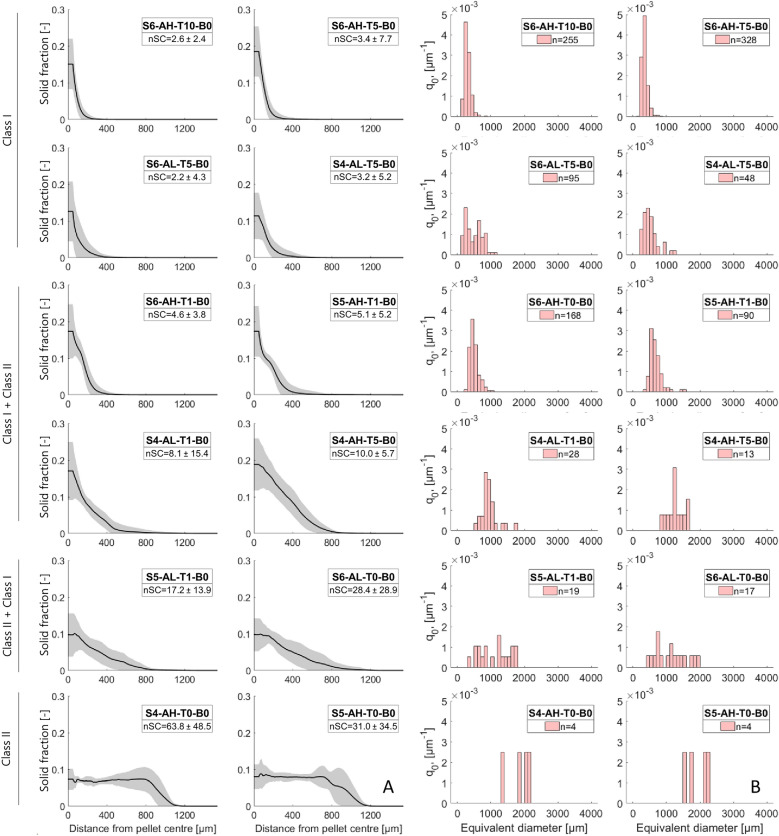
Pellet diameter distribution patterns are reflected in the solid fraction of individual pellets. **A** The solid fraction of selected population patterns is plotted against the radial coordinate of single pellets. The solid fraction was calculated for spherical shells with a width of 25 µm and an inner sphere of 50 µm, then averaged across the shells. Grey areas represent standard deviations, while ‘nSC’ represents the average number of spore clusters per pellet. **B** Pellet diameter distributions of the same populations, derived from 3D data with ‘*n*’ indicating the number of analysed pellets. The assignment of Class I, Class II and mixtures in the left margin is for orientation purposes and does not represent a strict categorisation

To analyse the internal structure of the pellets, the solid fraction over the pellet radius was calculated, describing the distribution of hyphae and talc. Selected cultivation conditions are presented in Fig. 7, including the average number of spore clusters per pellet. Previous studies [35] reported that pellets with a central spore core typically exhibit a solid fraction at the mass centre (y-axis section) of approx. 0.2. Notably, an increased number of spore clusters tends to reduce the solid fraction at the mass centre from above 0.2 to below 0.1. This suggests that in pellets with multiple spore clusters the centre of mass is not necessarily the densest region. Consequently, this criterion could be used to quantitatively differentiate pellet classes within a population based on their internal structure.

Comparing population patterns (Fig. 7) with the solid fraction reveals that cultivation conditions yielding similar diameter distributions also exhibit comparable solid fraction characteristics. As populations shift toward larger pellet diameters, the solid fraction within a 50 µm radius at the centre of mass increase in parallel with the number of spore clusters per pellet. This correlation can be explained by the positive relationship between the pellet diameter and the number of spore clusters within a population (see Figure S16), which in turn reduces the solid fraction at the mass centre. Conclusively, cultivation conditions that lead to smaller pellet diameters, such as those with a high spore concentration of 5 × 10^6^ spores mL^−1^ or high talc concentrations, had a reduced number of spore cores per pellet (Fig. 7). Interestingly, lower spore concentrations appear to result in a higher number of spores per pellet, likely due to the formation of fewer, but larger pellets. However, two populations, S6-AL-T5-B0 and S4-AL-T5-B0, deviate from this trend, exhibiting a lower hyphal fraction at the mass centre as expected based on their number of spore clusters per pellet and volume-equivalent pellet diameter.

Overall, populations with similar size distributions,—regardless of differences in cultivation conditions—tend to exhibit comparable solid fraction distributions within individual pellets.

Table S4 summarizes the key metrics derived from the cultivation experiments and SR-µ-CT analyses in this study. These data confirm that the mean total hyphal length, number of tips, and number of branches per pellet increased with pellet diameter. Notably, the pellets with the second smallest volume-equivalent diameter (S5-AL-T10-B0, 236 µm) showed the lowest average total hyphal length with just 3 ± 7 cm. Contrarily, the second largest pellets of nearly 2 mm diameter (S5-AH-T0-B0), sum up to a mycelial length of over 34 ± 18 m per pellet. These extremes, which are related to different cultivation conditions, also resulted in the fewest and highest number of tips and branches. The smallest pellets had an average of 215 ± 356 tips and 159 ± 293 branches per pellet, while the largest pellets reached an average of 210,170 ± 151,060 tips and 187,714 ± 91,880 branches per pellet. This increase of the number of tips, branches, and total hyphal length of roughly a factor of thousand increases proportional to the volume increase of the pellet and highlight the vast structural differences that can be achieved by different cultivation conditions.

## Discussion

For researchers working with filamentous organisms, the heterogeneity and flask-to-flask variance of pellet populations remain a significant challenge and often limit process development of submerged cultivations [12]. In this study we analysed pellet morphology at multiple levels to identify key influencing parameters and to provide a practical framework for selecting and achieving specific morphological outcomes.

The analysis of 48 cultivation conditions revealed a wide range of macromorphological pellet populations. Since dispersed mycelia constitute only a small fraction of shake flask cultivations [35] and were not visually detected in this study, image analysis focussed on pellet morphology. Populations with a similar distribution pattern were described previously for *Aspergillus* pellets [5, 8, 35, 51]. Our findings indicate that populations with larger average pellet diameters tend to exhibit higher heterogeneity and greater flask-to-flask variance. Given the multifactorial nature of pellet formation, it is likely that larger pellets are more susceptible to stochastic fluctuations during their development [13]. Linear regression confirmed that spore concentration, talc addition, and shaking frequency significantly reduce the pellet diameter, largely independently of one another. A recent study showed that spore agglomerates exceeding 200 µm in diameter rupture under shear stress during germination [35]. Given that talc microparticles are embedded in the spore agglomerates [50], it can be assumed that these three parameters, collectively affect the stability of the initial spore-talc aggregates under varying shear stress conditions, thereby contributing to the observed variance in pellet diameters. The breakage of spore-talc agglomerates may also explain the increased number of pellets, a phenomenon reported in other studies [16], but not explicitly measured here.

The 3D data suggest that a secondary agglomeration step plays a crucial role in the formation of co-agglomerative pellet morphologies. In *Streptomyces *spp., whose pellets form via hyphal-coagulative aggregation , the aggregation of germlings has been identified as a major factor contributing to heterogeneity in pellet populations [10]. In *A. niger,* spore(-talc) agglomerates appear to merge into larger structures after germination. We hypothesize that the timing of this process influences pellet morphology whereby early fusion leads to pellets with multiple spore cores which appear uniformly round, whereas later-stage fusion results in asymmetric or elongated pellets formed by the merging of mature or fragmented pellets. This breakage and agglomeration of pellets is a confirmation of earlier observations [35]. In conclusion, we suggest that the time after germination of spore aggregates and the secondary agglomeration seems to be critical and might be the driver for the formation of large (> 1000 µm) pellets. Our 3D imaging data indicate that there is a correlation between the pellet diameter and an increased number of spore clusters per pellet, suggesting that large pellets form via fusion of multiple spore aggregates. These multi-core pellets exhibit a more heterogeneous hyphal mass distribution across their radius, which is critical since oxygen transfer limitations are not solely dependent on the pellet diameter but also on the internal hyphal density [7, 14, 52].

It should be noted that the SR-µ-CT method used in this study also detected talc particles alongside the hyphae. As a result, metrics such as tip count, branch count, and total hyphal length must be interpreted with caution under high talc conditions, as these values might have been slightly overestimated due to the presence of talc. However, for solid fraction analysis across the pellet radius, the embedded talc was not considered a confounding factor; rather, it contributed to the overall density of the pellet, which is relevant for diffusion limitations. Recent studies have shown that it is possible to differentiate microparticles and mycelium, providing example images of embedded talc particles in dispersed mycelium [50]. Importantly, due to distinct differences in greyscale values and shape, spores were reliably distinguished from talc with the SR-µ-CT method used in this study.

Pellet morphology reproducibility remains a limiting factor in process development for filamentous organisms [45, 53]. Factors contributing to pellet heterogeneity likely also drive flask-to-flask variance. However, it remains unclear why some conditions yield highly reproducible pellet morphologies while others do not. In this study, the presence of baffles, lower spore concentrations, and larger pellet diameters were all associated with increased flask-to-flask variance.

In a productivity screen, five cultivation conditions resulting in reproducible macromorphological changes were used to show that larger pellets exhibited lower glucose consumption but higher total protein secretion based on the CDW. In all but one condition, pellet diameters exceeded 400 µm, suggesting that oxygen and nutrition limitations within the pellet did occur. An explanation for the differences in productivity could be that the smaller pellets use their metabolic capacities for biomass production, while larger ones have more capacity for protein production and secretion. This trade-off strategy was described previously in *A. niger*, showing that a higher biomass production led to a decreased glucoamylase production [54]. An explanation for this was given by [55], speculating that lower growth yields, as they appear under oxygen limited conditions, could lead to a higher availability of NADH and precursors for protein production. Another interesting influence of the pellet diameter on protein secretion was recently reported in [9]. Here, it was demonstrated that pellets of varying diameter exhibit a different secretome, which supposedly contribute differently to the development of the whole population and could even be beneficial to stress survival.

While larger pellets seem additionally to be promising in terms of rheological properties, they present challenges for applications such as lower reproducibility and often negatively associated greater heterogeneity. For tailored seed culture populations, genetic engineering could offer an option to overcome some of these challenges. Potential targets include genes involved in the secondary agglomeration step, e.g. those encoding synthesizing enzymes for glycoproteins or outer glucans on the fungal cell wall [56]. An overview for the control and impact of small and large pellet populations is summarised in Table 2.

**Table 2:** Summary of pellet population phenotypes of *A. niger* observed in this study

	Small pellet diameter population	Large pellet diameter population
Inoculation and cultivation parameter	High spore concentration High talc concentration High rpm	Low spore concentration Low talc concentration Low rpm
Culture characteristics	Low heterogeneity High viscosity Fast glucose consumption Fast biomass accumulation Low flask-to-flask variance	High heterogeneity Low viscosity Low glucose consumption High protein secretion High flask-to-flask variance
Pellet characteristics	Predominantly Class I Total hyphal length in cm range ~ 200 tips (diameter ~ 200 µm) ~ 200 branches (diameter ~ 200 µm) Number of spore cluster < ~ 5	Predominantly Class II Total hyphal length in m range ~ 200,000 tips (diameter ~ 2 mm) ~ 200,000 branches (diameter ~ 2 mm) Number of spore cluster > ~ 10

For details, see text

## Conclusion

In this study, we demonstrate that the pellet diameter and population heterogeneity of *A. niger* can be altered through process parameters, allowing for the cultivation of reproducible pellet populations with profound impacts on productivity. The extensive morphological dataset allowed us to show the independent influence of three parameters significantly reducing pellet diameter, and to offer a tool for median pellet diameter prediction. Additionally, we introduce a classification system for pellets of *A. niger* based on their internal architecture. Pellet morphology is shaped by the distinct agglomeration steps occurring at different growth stages—initially at an early growth stage through accumulation of spores, and later via cell aggregation [35]. We hypothesize that, depending on the growth phase, pellets develop with either a single or multiple spore centres. In addition, agglomeration at a later stage can lead to the formation of asymmetrical pellet structures. However, the development of pellet formation remains a multifactorial process, as no single parameter can fully capture the complexity of heterogeneous pellet development of the filamentous fungus *A. niger*. Still, modelling approaches are fundamental to uncover limitations and can target further research requirements. Over all, this study highlights the need to expand the concept of coagulative pellet formation, particularly for pellets originating from a central spore core, and to incorporate a more comprehensive understanding of micromorphological variability.

## Supplementary Information

Supplementary Material 1.

Supplementary Material 2.

## Data Availability

The data sets used and/or analysed during the current study are available from the corresponding author on reasonable request.
