# Supplementary material for: Adjusting Aspergillus niger pellet diameter, population heterogeneity, and core architecture during shake flask cultivation
Source: Biotechnol Biofuels Bioprod. 2025 Jun 12;18:62. doi: 10.1186/s13068-025-02661-2 (PMC12160380; doi:10.1186/s13068-025-02661-2)
Supplement: Supplementary file 1 — Supplementary Material 1. [file 13068_2025_2661_MOESM1_ESM.pdf]

Supplementary Information to

Adjusting *Aspergillus niger* pellet diameter, population heterogeneity, and core architecture during shake flask cultivation

Engelbert, K., Deffur, C., Cairns, T. C., Zhang, F., Kheirkhah, T., Winter, H., Junne, S., Neubauer, P., Briesen, H., Meyer, V.

Correspondence:

Karin Engelbert (karin.engelbert@tu-berlin.de), Technische Universität Berlin, Institute of Biotechnology, Chair of Applied and Molecular Microbiology, Straße des 17. Juni 135, 10623 Berlin, Germany

Vera Meyer (vera.meyer@tu-berlin.de), Technische Universität Berlin, Institute of Biotechnology, Chair of Applied and Molecular Microbiology, Straße des 17. Juni 135, 10623 Berlin, Germany

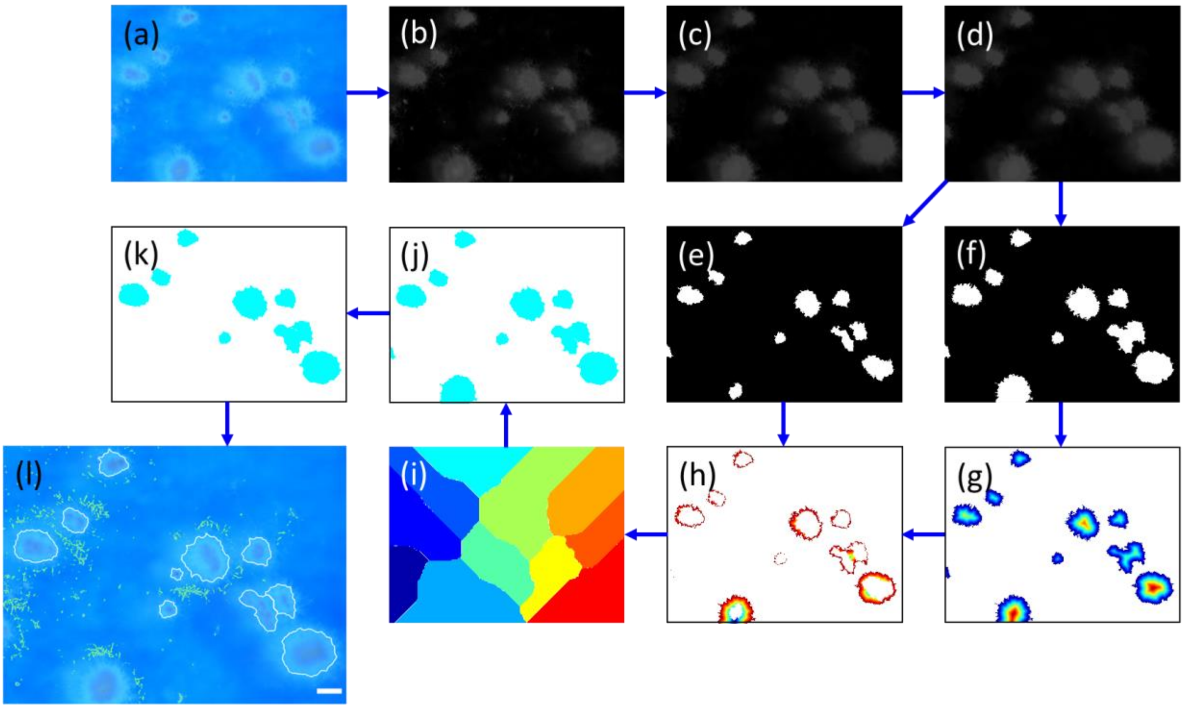

Figure S1 Overview of the image analysis and data processing (images a-k) for pellet segmentation without the need to exhibit clear spore agglomerates, suited for densely grown pellets. Image (l) shows the result of pellet segmentation (white) and mycelium detection (green). The scale bar of image (l) represents a length of 500 µm.

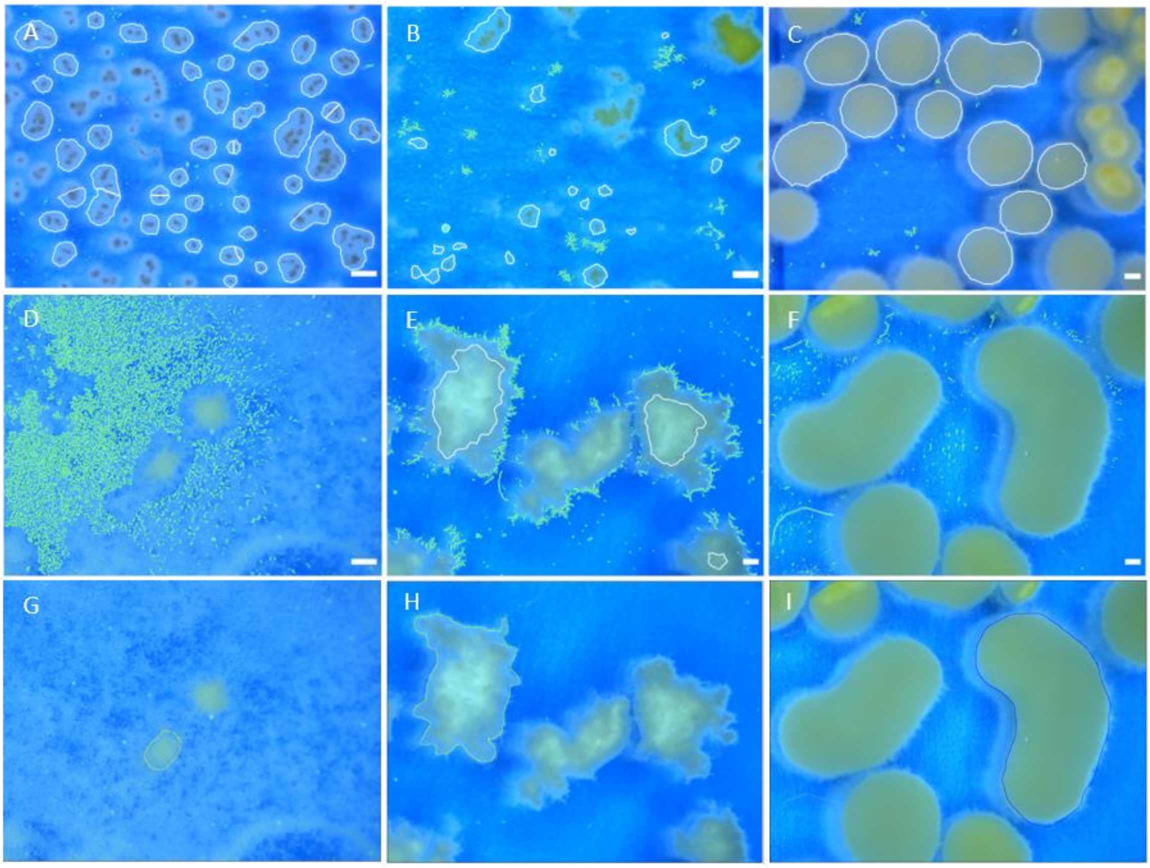

Figure S2 Example images for representative image analysis performed with the adjusted image analysis tool of different pellets types (A-C). Examples for not representative image analysis because of e.g. the presence of talcum (D), irregular shapes (E) or exceeding pellet size (F) and their examples of manual analysis with Fiji ImageJ (H-J). The scale bar represents a length of 250 µm.

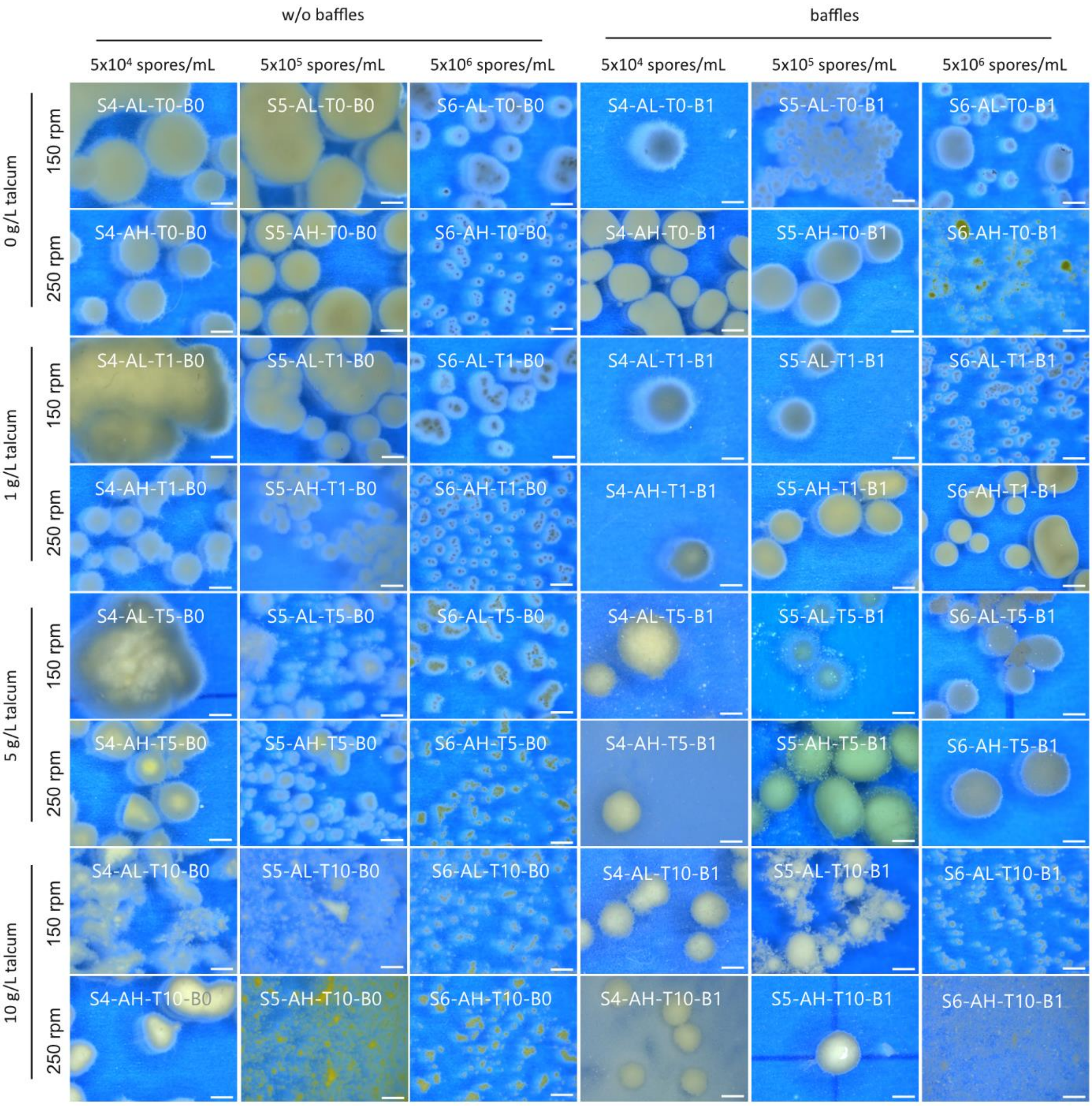

Figure S3a Exemplary stereomicroscopic images of 48 cultivation conditions. *A. niger* was cultivated in shake flasks with cultivation parameters varied in inoculation spore concentration (S), shaking frequency (A), different talc concentrations (T) and flask form (B). The images show replicate ‘a’ of biological triplicates for each condition; these correspond to the blue populations shown in Figure 2. Images were taken after 24 h, the scale bar represents 1000 μm.

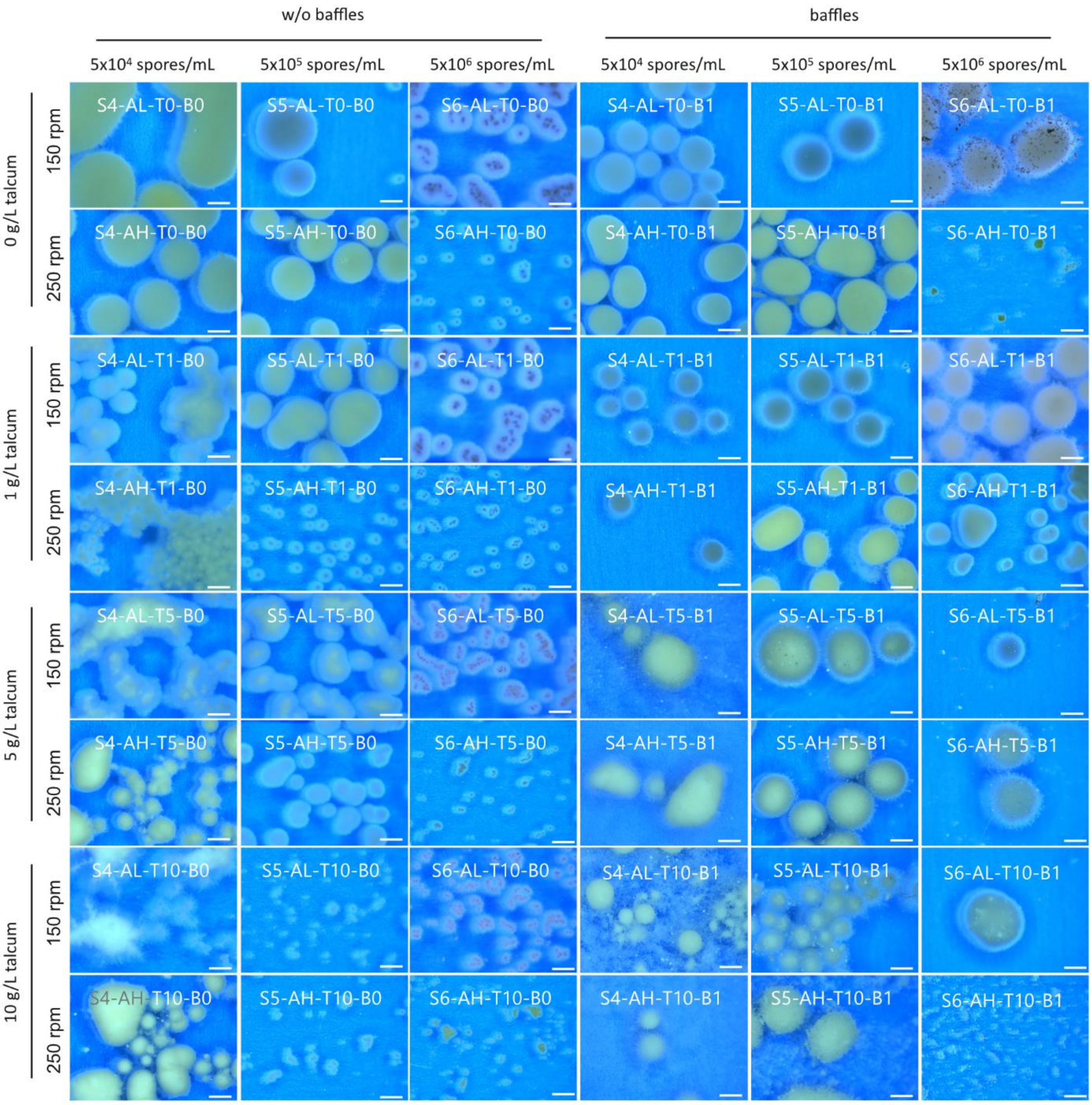

Figure S3b Exemplary stereomicroscopic images of 48 cultivation conditions. *A. niger* was cultivated in shake flasks with cultivation parameters varied in inoculation spore concentration (S), shaking frequency (A), different talc concentrations (T) and flask form (B). The images show replicate 'b' of biological triplicates for each condition; these correspond to the red populations shown in Figure 2. Images were taken after 24 h, the scale bar represents 1000 µm.

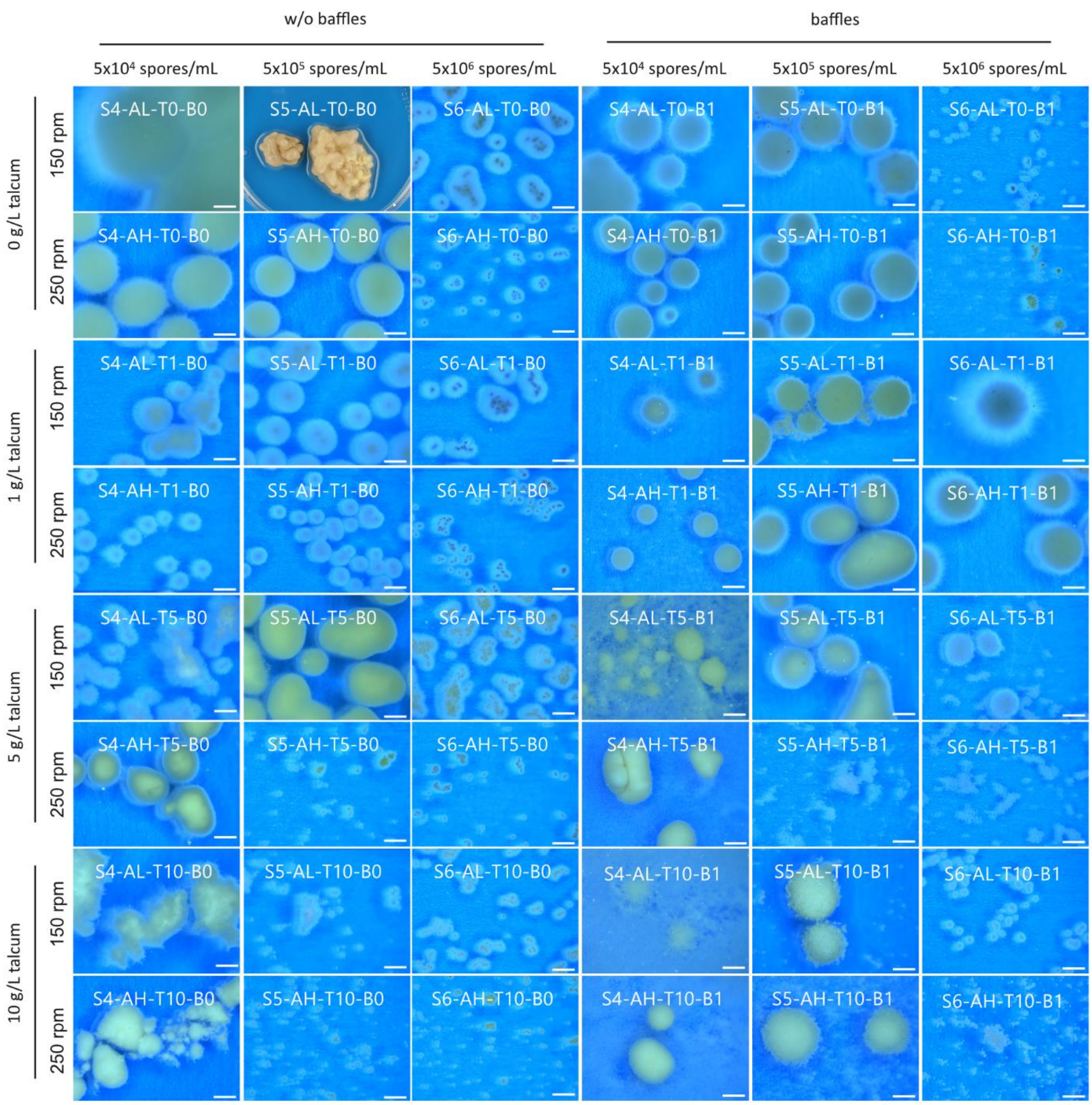

Figure S3c Exemplary stereomicroscopic images of 48 cultivation conditions. *A. niger* was cultivated in shake flasks with cultivation parameters varied in inoculation spore concentration (S), shaking frequency (A), different talc concentrations (T) and flask form (B). The images show replicate 'c' of biological triplicates for each condition; these correspond to the yellow populations shown in Figure 2. Images were taken after 24 h, the scale bar represents 1000 µm.

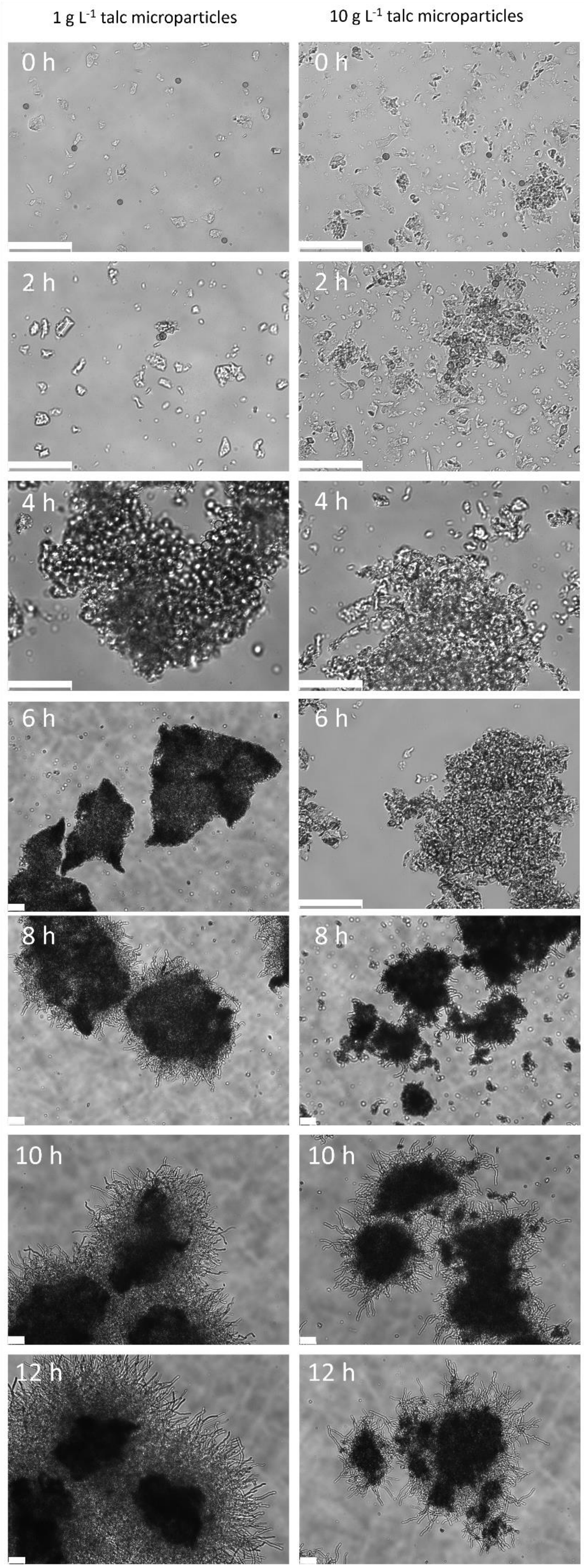

Figure S4 Integration of talcum particles into pellets over 12 h. Condition S6-AL-T10-B0 (1 g L^{-1} talcum) and S6-AL-T1-B0 (10 g L^{-1} talcum) were exemplary chosen to follow the pellet formation process. The images were taken using a Leica DM 5000 CS DIC microscope, equipped with a DFC365 FX CCD-Microscopy camera (Leica Microsystems GmbH). Scale bar represents 50 µm.

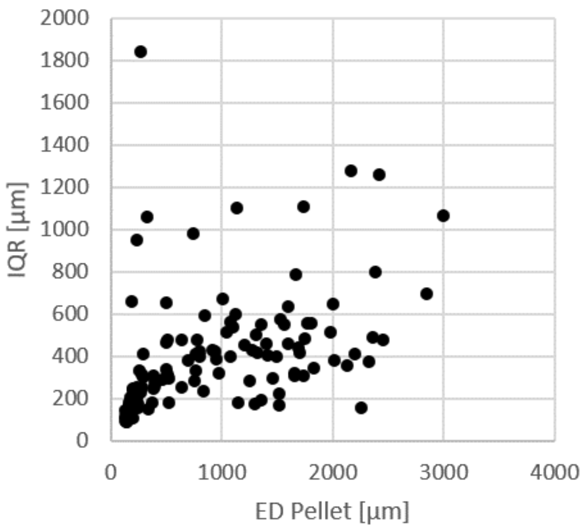

Figure S5 Pellet diameter heterogeneity increases with average pellet diameter. The interquartile range (IQR) showing the spread of the inner 50 % of the population was plotted against the median of each population with at least 30 analyzed pellets. Each data point represents one shake flask culture.

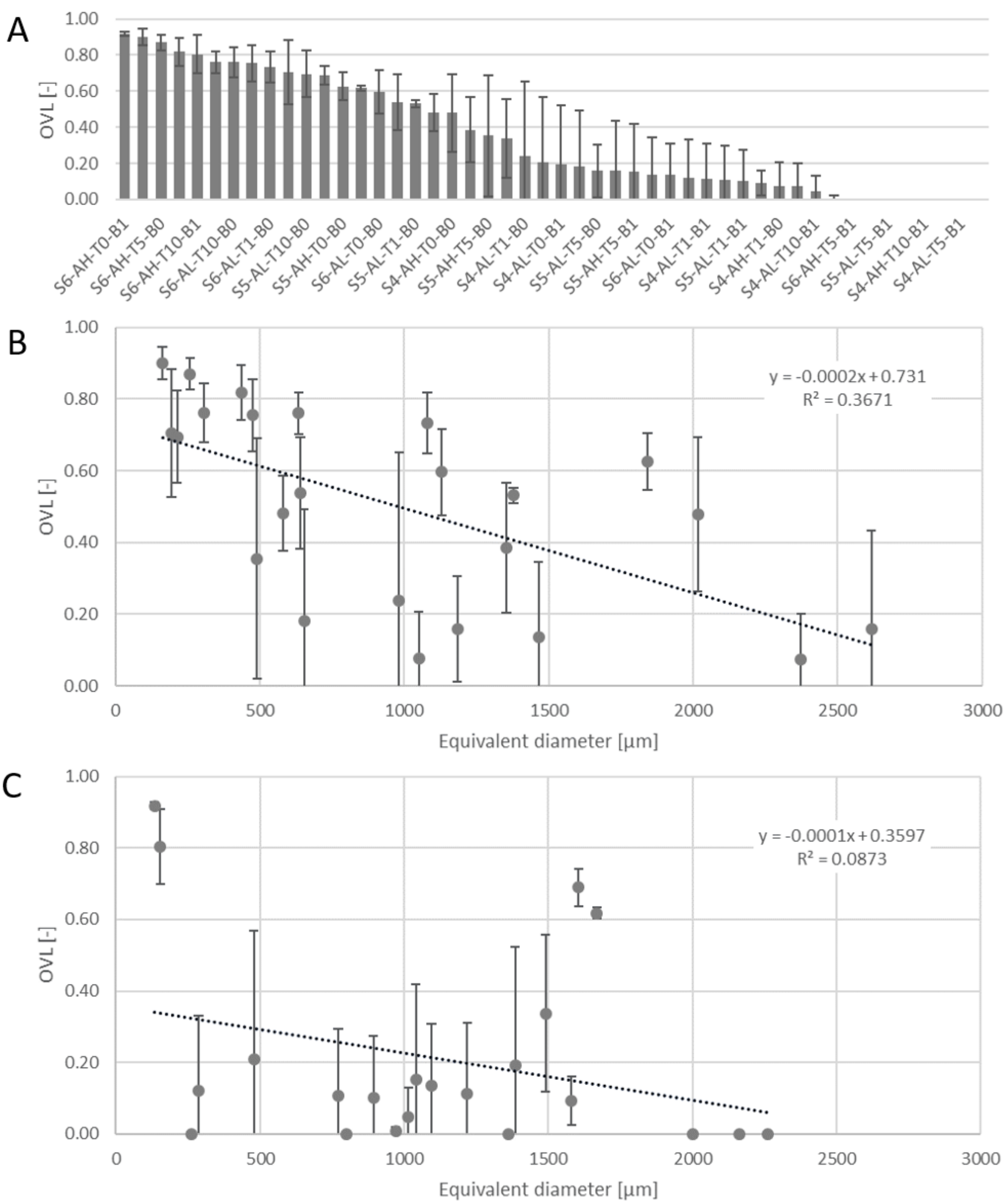

Figure S6 Flask-to-flask variance of culture conditions measured by the overlap coefficient (OVL) of the pellet density distributions (q_0) of replicates (n=3). (A) OVL of 48 cultivation conditions. OVL plotted against median pellet diameter of cultivation conditions with non-baffled (B) and baffled (C) flasks.

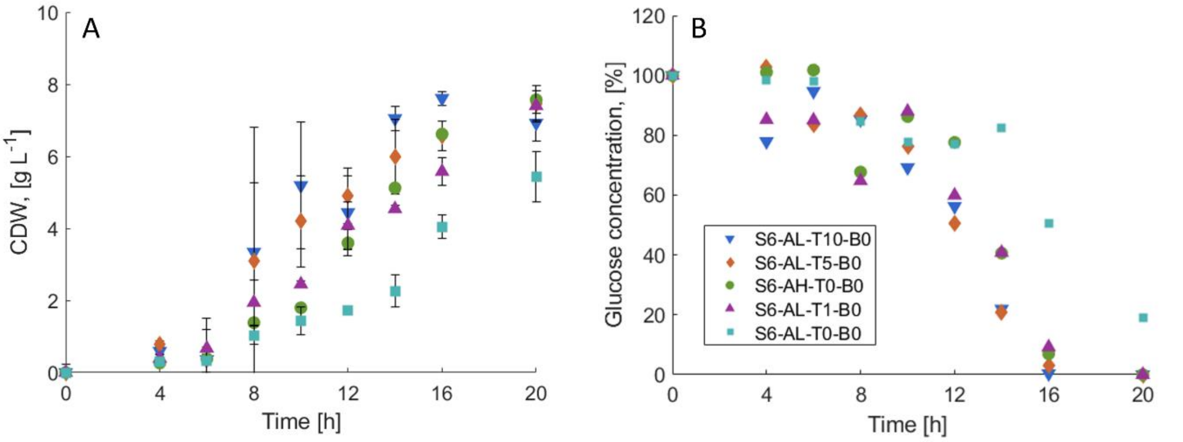

Figure S7 Growth behaviour of five populations with different pellet diameters. One shake flask was harvested for each time point. To determine the cell dry weight (CDW), the flasks were sampled three times and the percentage by weight of the talc was subtracted from the biomass (A). Normalised glucose concentration (B).

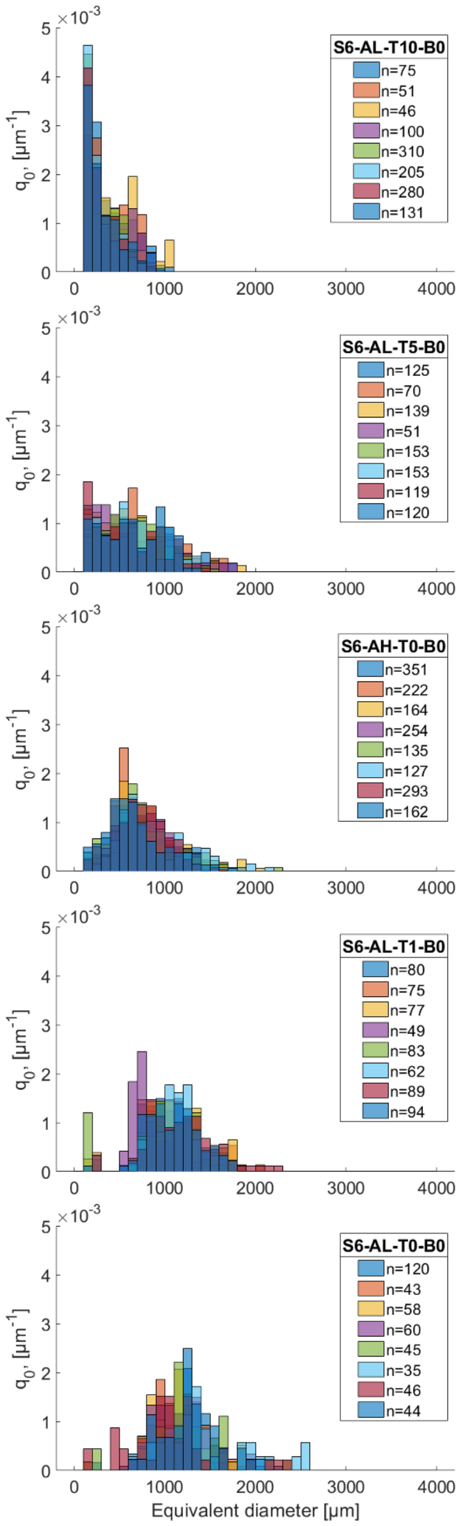

Figure S8 Pellet diameter distributions of five cultivation conditions of *A. niger* with low flask-to-flask variance. Cultures were grown in multiple replicates (n=8) for 16 h. The x-axis shows the measured area equivalent pellet diameter and the y-axis the number-density distribution of the pellet populations (q_0), describing the number of pellets per bin, normalised to the total pellet number of analysed pellets 'n'. The bin size is 100 μm.

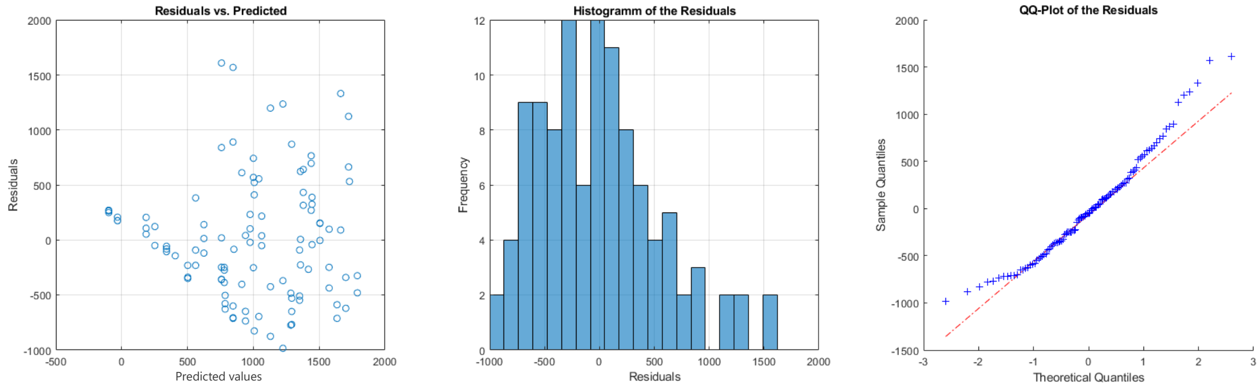

Figure S9: Graphs for visual assessments of multiple linear regression. The residual plot (left) shows the differences between the predicted and actual values. The observed funnel shape indicates heteroscedasticity. The histogram (middle) and QQ-plot (right) indicate a skewed distribution of the residuals, deviating from normality. The presented graphs are based on a multiple linear regression performed using the MATLAB function 'fitlm' to predict the median pellet diameter (PD, µm) based on the independent variables: spore concentration (S), agitation frequency (A), talc concentration (T), and presence of baffles (B). The analysis included 107 observations. The model used was PD ~ 1 + S + A + T + B. Among the independent variables, S, A, and T are significant (*p-value* < 0.05), while B is not significant (*p-value* > 0.05). The adjusted R^2 value is 0.41.

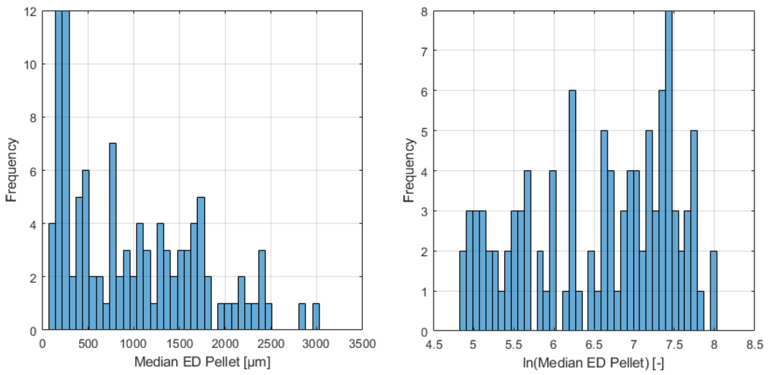

Figure S10: Histograms of the median equivalent pellet diameter (ED) distribution from 107 populations derived by different cultivation conditions. The histogram on the left shows a highly-skewed distribution, results from numerous cultivation conditions leading to the formation of pellets with smaller pellet diameter. Applying a natural logarithmic transformation (ln) to these observations (right), which serves as the depended variable for the multiple linear regression, results in a more evenly distribution.

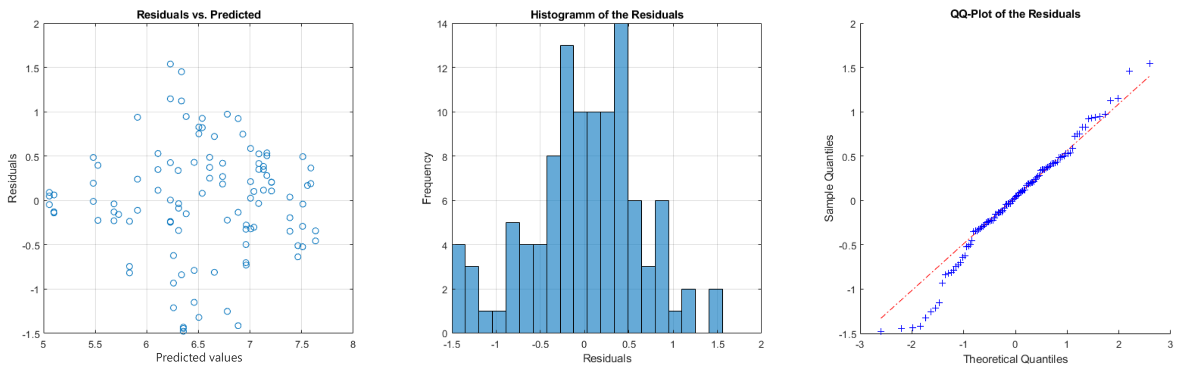

Figure S11: Graphs for visual assessments of multiple linear regression after applying a natural logarithmic transformation (ln) to the dependent variable. The residual plot (left) shows a more randomised distribution of the residuals compared to the non-transformed multiple linear regression. The histogram (middle) and QQ-plot (right) indicate a more evenly distribution of the residuals. Together this indicate an improvement in fulfilling the assumptions for multiple linear regression. The presented graphs are based on a multiple linear regression performed using the MATLAB function 'fitlm' to predict the median pellet diameter (PD, µm) based on the independent variables: spore concentration (S), agitation frequency (A), talc concentration (T), and presence of baffles (B). The analysis included 107 observations. The model used was ln(PD) ~ 1 + S + A + T + B. Among the independent variables, S, A, and T are significant (*p-value* < 0.05), while B is not significant (*p-value* > 0.05). The adjusted R^2 value is 0.51.

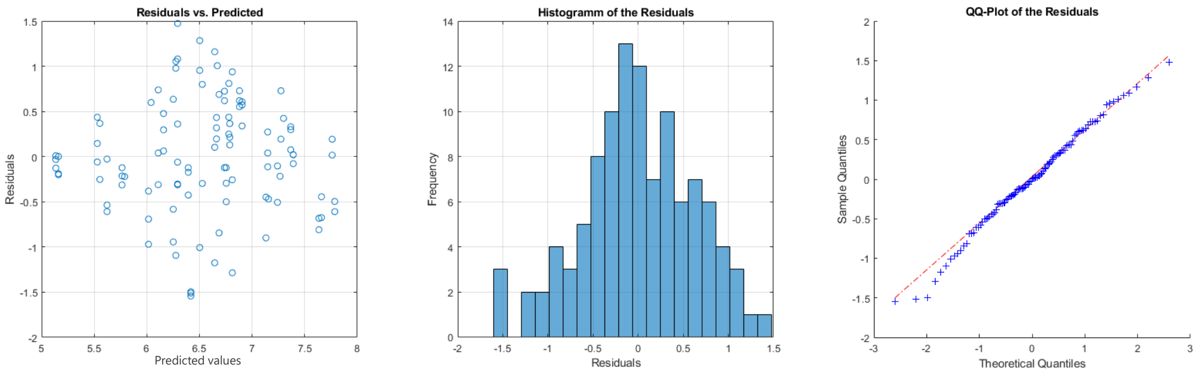

Figure S12: Graphs for visual assessments of multiple linear regression after applying a natural logarithmic transformation (ln) to the independent variable. The residual plot (left) shows a more randomised distribution of the residuals compared to the non-transformed multiple linear regression. The histogram (middle) and QQ-plot (right) indicate a more normally distribution of the residuals. Together this indicate an improvement in fulfilling the assumptions for multiple linear regression. The presented graphs are based on a multiple linear regression performed using the MATLAB function 'fitlm' to predict the median pellet diameter (PD, µm) based on the independent variables: spore concentration (S), agitation frequency (A), talc concentration (T), and presence of baffles (B). The analysis included 107 observations. The model used was ln(PD) ~ 1 + ln(S)+ ln(A) + T + B. Among the independent variables, S, A, and T are significant (*p-value* < 0.01), while B is not significant (*p-value* > 0.05). The adjusted R^2 value is 0.51 (Table S1).

Table S1 Estimate coefficients from multiple linear regression. MATLAB function 'fitlm' was used to predict the median pellet diameter (PD, µm) based on the independent variables: spore concentration (S), agitation frequency (A), talc concentration (T), and presence of baffles (B). The analysis included 107 observations. The model used was ln(PD) ~ 1 + ln(S)+ ln(A) + T + B. Among the independent variables, S, A, and T are significant (*p-value* < 0.01), while B is not significant (*p-value* > 0.05). The adjusted R^2 value is 0.51.

	**Estimate**	**SE**	**tStat**	***p-value***
(Intercept)	13.911	1.3583	10.241	2.3795e-17
S	-0.21128	0.033194	-6.3651	5.6439e-09
A	-0.77126	0.24485	-3.1499	0.0021432
T	-0.12583	0.015848	-7.9394	2.7638e-12
B	0.024477	0.12959	0.18888	0.85057

Table S2 Correlation matrix estimate coefficients of significant independent variables. MATLAB function 'corr' was used to calculate the pairwise linear correlation coefficient between each pair of variables spore concentration (S), agitation frequency (A) and talc concentration (T). S and A have been used after being transformed using the natural logarithm (ln).

	**ln(S)**	**ln(A)**	**T**
**ln(S)**	1.0000	0.0364	0.0041
**ln(A)**	0.0364	1.0000	−0.0845
**T**	0.0041	−0.0845	1.0000

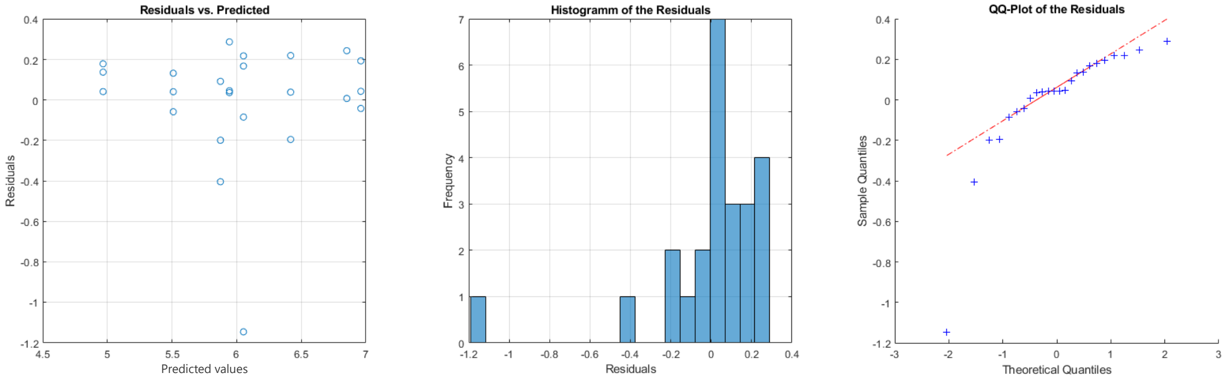

Figure S13: Graphs for visual assessments of multiple linear regression with a reduced dataset of 24 observations. The residual plot (left) shows a mostly randomised distribution of the residuals around the zero line. The histogram (middle) and QQ-plot (right) indicate the normally distribution of the residuals. Together this indicate an improvement in fulfilling the assumptions for multiple linear regression. The presented graphs are based on a multiple linear regression performed using the MATLAB function 'fitlm' to predict the median pellet diameter (PD, µm) based on the independent variables: agitation frequency (A) and talc concentration (T). The model used was ln(PD) ~ 1 + ln(A) + T. The adjusted R^2 value is 0.80.

Table S3 Estimate coefficients from multiple linear regression. MATLAB function 'fitlm' was used to predict the median pellet diameter (PD, µm) based on the independent variables agitation frequency (A) and talc concentration. The analysis included 24 observations of non-baffled flasks and highest spore concentration of $5x10^6$ spores mL^{-1}. The model used was ln(PD) ~ 1 + ln(A) + T. The adjusted R^2 value is 0.80.

	**Estimate**	**SE**	**tStat**	***p-value***
(Intercept)	15.863	1.3067	12.14	5.8808e-11
ln(A)	-1.7769	0.24572	-7.2311	4.0003e-07
T	-0.10856	0.015739	-6.8974	8.1406e-07

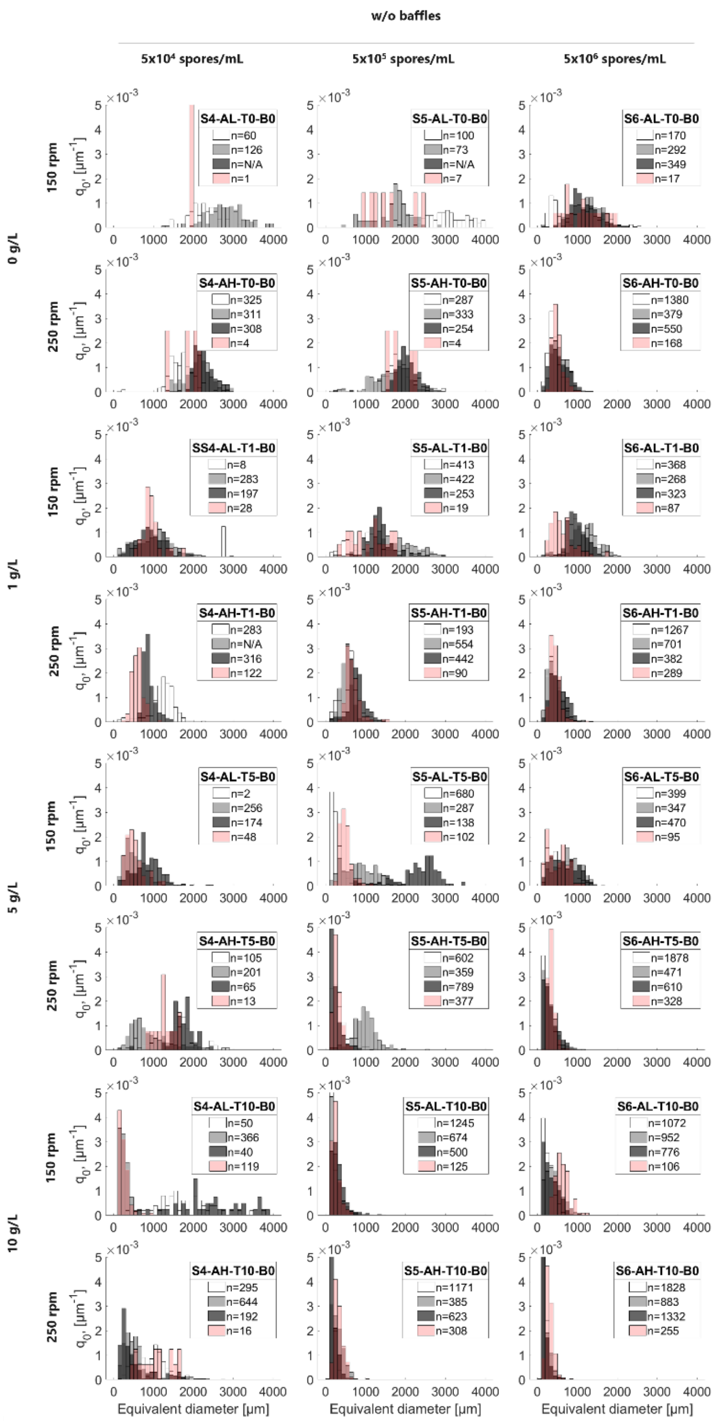

Figure S14 Comparison of pellet size populations derived from 2D and 3D measurements. Pellet diameter distributions from a fourth replicate (red) measured by SR-μCT are compared to the sizes derived from 2D image analysis triplicates (grey). The x-axis shows the measured equivalent pellet diameter and the y-axis the normalized number density of pellets, in the population, having this size. All replicates were grown from independent spore solutions with 'n' being the number of analysed pellets for each condition and N/A for populations that could not be analysed. To enhance readability, the highest values have been partially cropped (x- and y-axis), the bin size is 100 μm.

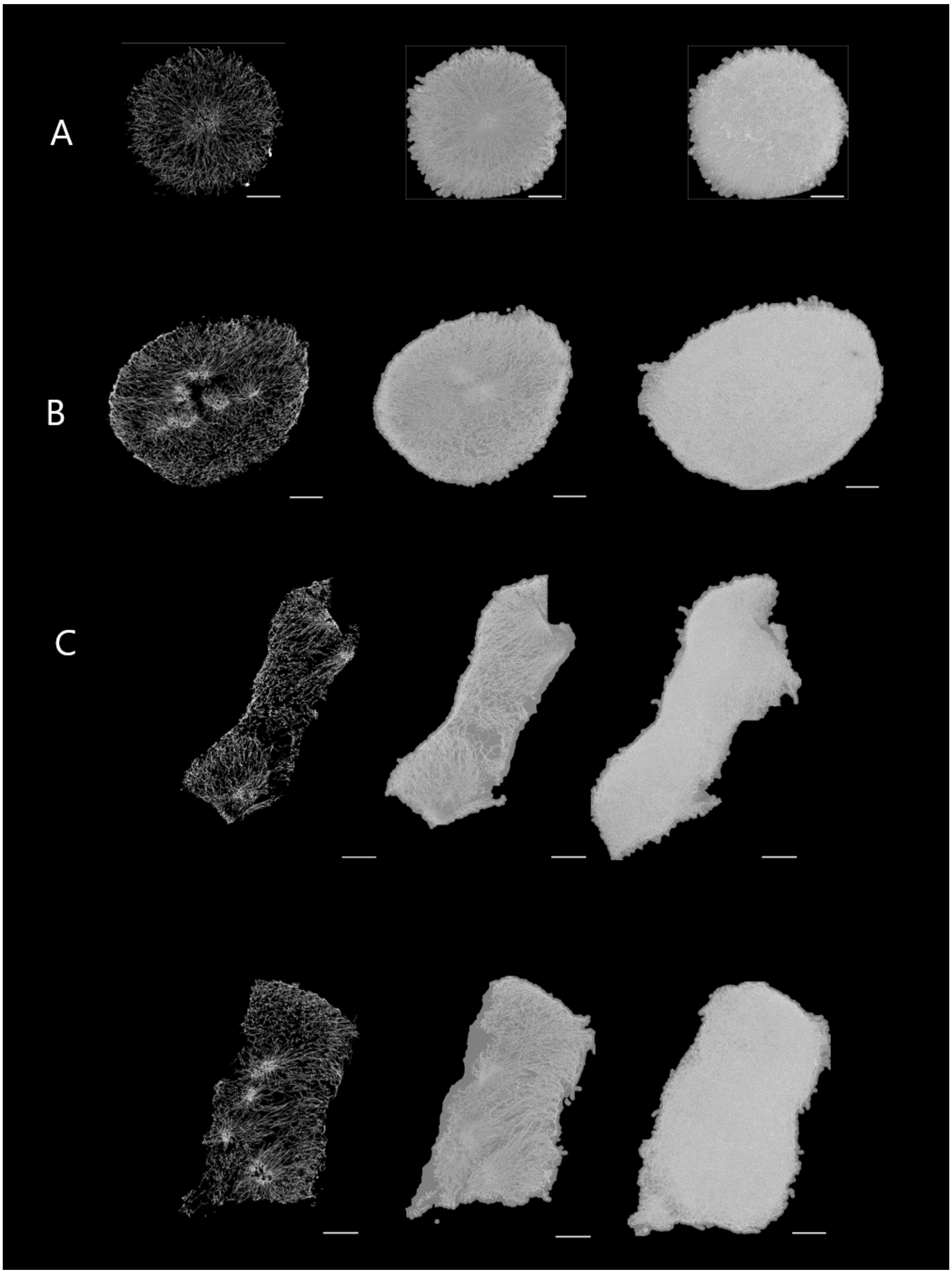

Figure S15 Pellets that appears to derive from several thorn pellet fragments. Four exemplary pellets are shown in different projections. On the left the mean intensity projection is shown, which represents the mean grey value of a 25 µm slice around the pellet's mass centre. In the middle the max intensity projection, the cumulative grey values of a 50 µm slice around the mass centre is given, and on the right the cumulative grey values of the whole pellet, giving an impression of the outer structure. The scale bar represents 250 µm. Shown are a single spore core pellet (A), a multi spore core pellet (B) and two irregular shaped pellets fused together by "thorn" pellets (C).

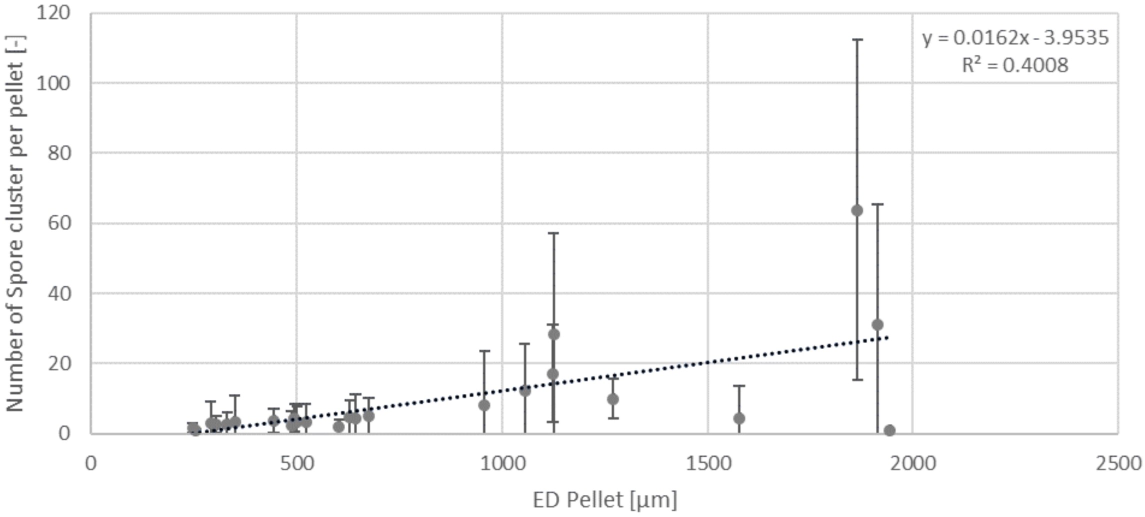

Figure S16 Relation of spore clusters and pellet diameter. The number of spore cluster per pellet analysed from 3D data by the MATLAB function ‘dbscan’, was plotted against the average pellet diameter of the population.

Table S4 Quantitative analysis of submerged cultivations using SR-µ-CT.

	Spore conc. [spores/mL]	Talc [g/L]	Shaking frequency [rpm]	Mean ED pellet [µm]	Median ED pellet [µm]	Porosity [-]	Hyphal diameter [µm]	Total hyphal length (THL)/ pellet [m]	Number of tips/ pellets [-]	Number of branches/ pellet [-]	Hyphal branch unit (HBU) [-]	Hyphal growth unit (HGU) [-]	Number of spore cluster/ pellet [-]	Number of spores/ pellet [-]
S4-AL-T10-B0 (n = 119)	50000	10	150	247±127	218	0.91±0.07	4.41±0.44	0.07±0.19	288±789	252±68	269±101	257±188	1.6±1.3	575±1298
S5-AL-T10-B0 (n = 125)	500000	10	150	255±105	236	0.97±0.01	3.5±0.19	0.03±0.07	215±356	159±293	195±33	133±42	1.1±0.3	179±761
S5-AH-T5-B0 (n = 377)	500000	5	250	291±100	271	0.93±0.02	3.37±0.14	0.13±0.15	993±1139	577±666	224±23	131±32	2.9±6.4	2294±4492
S6-AH-T10-B0 (n = 255)	5000000	10	250	304±92	291	0.95±0.02	3.36±0.18	0.09±0	658±607	410±404	217±19	137±33	2.6±2.4	1170±1509
S5-AH-T10-B0 (n = 308)	500000	10	250	332±112	307	0.95±0.02	3.48±0.2	0.13±0.15	849±1033	587±675	219±23	159±44	2.7±3.5	1501±2156
S6-AH-T5-B0 (n = 328)	5000000	5	250	350±87	329	0.94±0.01	3.18±0.14	0.19±0	1649±1579	903±884	209±15	114±17	3.4±7.7	2574±4515
S6-AH-T1-B0 (n = 289)	5000000	1	250	444±126	424	0.95±0.02	3.21±0.13	0.33±0.33	2050±2123	1700±1692	196±14	169±39	3.7±3.3	1498±1332
S6-AL-T5-B0 (n = 95)	5000000	5	150	489±248	439	0.96±0.03	3.52±0.3	0.42±0	1685±2162	1737±2062	237±34	250±100	2.2±4.3	3830±9280
S6-AH-T0-B0 (n = 168)	5000000	0	250	495±134	478	0.92±0.02	3.34±0.14	0.61±0.5	3330±2655	3124±2636	198±17	189±55	4.6±3.8	3707±3043
S4-AL-T5-B0 (n = 48)	50000	5	150	523±229	481	0.96±0.01	3.54±0.27	0.48±0.86	2487±4134	2198±3736	206±20	174±31	3.2±5.2	2972±5986
S5-AL-T5-B0 (n = 102)	500000	5	150	501±147	483	0.97±0.01	3.45±0.19	0.29±0.38	1452±1912	1504±1920	190±14	202±44	3.3±4.7	1772±3079
S6-AL-T1-B0 (n = 87)	5000000	1	150	643±305	554	0.97±0.01	3.48±0.2	0.7±1.25	2567±4110	3524±6299	199±24	249±69	4.3±6.8	1785±3193
S4-AH-T1-B0 (n = 122)	50000	1	250	603±131	597	0.96±0.01	3.49±0.14	0.52±0.38	3132±2131	2620±1889	200±12	167±35	2.1±2	1105±1059
S6-AL-T10-B0 (n = 106)	5000000	10	150	630±174	606	0.95±0.01	3.26±0.06	0.87±0	4600±4130	4002±3412	213±12	193±40	4.7±4.7	7875±8259
S5-AH-T1-B0 (n = 90)	500000	1	250	676±188	628	0.95±0.01	3.36±0.07	1.19±1.91	6315±9832	6434±10080	185±8	189±34	5.1±5.2	2419±2803
S4-AL-T1-B0 (n = 28)	50000	1	150	958±236	922	0.96±0.01	3.49±0.13	2.58±2.67	11659±10164	12838±12830	199±9	215±51	8.1±15.4	4940±6925
S4-AH-T10-B0 (n = 16)	50000	10	250	1055±416	1051	0.91±0.03	3.43±0.09	10.21±10.66	60124±65186	37045±3862	264±24	166±24	12.4±13.1	27048±22144
S6-AL-T0-B0 (n = 17)	5000000	0	150	1126±472	1111	0.92±0.02	3.32±0.08	9.37±9.73	46130±58490	48293±49640	196±16	258±131	28.4±28.9	21344±21480
S4-AH-T5-B0 (n = 13)	50000	5	250	1271±248	1240	0.94±0.01	3.27±0.04	9.18±5.49	45122±25807	38823±22315	233±12	201±25	10.0±5.7	87129±50006
S5-AL-T1-B0 (n = 19)	500000	1	150	1124±463	1274	0.95±0.01	3.46±0.1	5.28±4.73	15540±14431	27253±24513	192±9	327±86	17.2±13.9	6293±6056
S5-AL-T0-B0 (n = 7)	500000	0	150	1577±565	1479	0.95±0	3.29±0.04	15.73±15.12	84750±98864	84750±82610	188±8	195±37	4.5±9.1	1235±2770
S5-AH-T0-B0 (n = 4)	500000	0	250	1914±307	1914	0.93±0.01	3.28±0.05	33.99±18.04	210170±151060	187714±91880	179±9	180±41	31.0±34.5	10161±13879
S4-AL-T0-B0 (n = 1)	50000	0	150	1944	1944	0.96	3.53	16.23	55320	86626	187	293	1	115
S4-AH-T0-B0 (n = 4)	50000	0	250	1865±355	1946	0.93±0.01	3.34±0.07	29.97±13.86	183674±110820	160307±75615	188±5	173±29	63.8±48.5	27498±22781

Mean values, standard deviation and number of pellets analysed per culture condition are given (n). ED pellet = Equivalent pellet diameter.
